# Anti-SSTR2 peptide based targeted delivery of potent PLGA encapsulated 3,3’-diindolylmethane nanoparticles through blood brain barrier prevents glioma progression

**DOI:** 10.18632/oncotarget.18689

**Published:** 2017-06-27

**Authors:** Arijit Bhowmik, Sayak Chakravarti, Aparajita Ghosh, Rajni Shaw, Suman Bhandary, Satyaranjan Bhattacharyya, Parimal C. Sen, Mrinal K. Ghosh

**Affiliations:** ^1^ Signal Transduction in Cancer and Stem Cells Laboratory, Translational Research Unit of Excellence (TRUE), Division of Cancer Biology and Inflammatory Disorder, Council of Scientific and Industrial Research-Indian Institute of Chemical Biology (CSIR-IICB), Kolkata 700091, India; ^2^ Division of Molecular Medicine, Bose Institute, Centenary Campus, Kolkata 700054, India; ^3^ Division of Surface Physics, Saha Institute of Nuclear Physics, Kolkata 700064, India

**Keywords:** glioma, blood brain barrier, 3,3’-diindolylmethane encapsulated nanoparticle, somatostatin receptor 2 peptide, epidermal growth factor receptor

## Abstract

Current therapy for Glioblastoma is insufficient because of the presence of blood brain barrier. It limits the transport of essential drugs to the tumor sites. To overcome this limitation we strategized the delivery of an anticancer compound 3,3’-diindolylmethane by encapsulation in poly (lactic-co-glycolic acid) nanoparticles. These nanoparticles were tagged with a novel peptide against somatostatin receptor 2 (SSTR2), a potential target in glioma. The nanoformulation (27-87nm) had loading and encapsulation efficiency of 7.2% and 70% respectively. It was successfully internalized inside the glioma cells resulting in apoptosis. Furthermore, an *in vivo* bio-distribution study revealed the selective accumulation of the nanoformulation into rat brain tumor sites by crossing the blood brain barrier. This resulted in abrogation of epidermal growth factor receptor pathway activation in glioma cells. Our novel nanopreparation therefore shows great promise to serve as a template for targeted delivery of other therapeutics in treating GBM.

## INTRODUCTION

Among different types of brain tumor diagnosed till date, malignant glioma remains a significant clinical challenge with an occurrence of around 45%- 50%, which makes it more aggressive, often causing death of victims within one-two years of diagnosis [1, 2]. The highly infiltrative nature of the malignant glioma cells makes it almost impossible to cure even by surgery or radiotherapy. Available drug therapies are also less potent with adverse side effects resulting in a very poor prognosis rate [3]. Furthermore, a major hurdle in the therapeutic intervention of malignant gliomas is the presence of the BBB that confers resistance to the delivery of efficient drugs to the brain tumour site [4]. Local drug administration like intracerebral implants causes very little benefit to the patients with gliomas [5]. This is most likely due to the restricted diffusion of the drugs in brain tissue. Intracarotid infusions of hypertonic mannitol may cause temporary disruption of the BBB, which has been used with few successes; however, highly complex surgical procedures and toxic side effects limit its utility [6]. Therefore, there is an urgent need to develop a new therapeutic strategy to overcome the problems related to BBB and to improve the survival chances of GBM patients.

An ideal way of addressing the present therapeutic challenges of GBM could be the use of brain penetrating polymeric nanoparticles that can be differentially taken up by glioma cells and release their payload more efficiently in a sustained manner to achieve a significant clinical response. Poly(lactic-co-glycolic acid) (PLGA), a biocompatible polymer, has been involved in various drug delivery applications and assures successful tumor targeting. Moreover, surface modifications of such drug loaded PLGA nanoparticles via linking with specific ligands targeting specific receptors that remain overexpressed in the diseased cells could prove to be a novel strategy in GBM therapeutics.

In the recent past, phytochemicals have attracted much attention as potential chemopreventive agents as these natural compounds produce antitumor effects in preclinical models and humans [7]. One of these compounds which has found a significant use in human is 3,3’ Diindolylmethane (DIM), produced from the digestion of Indole-3-Carbinol (I3C) in cruciferous vegetables like Brussels sprouts, broccoli, cauliflower, kale etc. [8]. DIM has been reported to inhibit proliferation and induce programmed cell death in many types of cancer cells including breast, endometrium, colon, and prostate [9–12]. However, low oral bioavailability and hydrophobicity of DIM limit its passage through the tight junctions of the brain, thereby displaying poor anticancerous effects against high grade brain cancers [13–16]. In the present study we developed PLGA nanoencapsulated DIM with an aim to attain the following advantages: (i) to surmount the drug solubility problem, (ii) to enhance passaging of drugs selectively across the BBB, (iii) sustained drug release and (iv) to achieve high biocompatibility and tumor targeting efficiency. In order to augment tumor targetability, we synthesized a novel synthetic peptide against Somatostatin receptor 2 (SSTR2) which is a member of the G protein-coupled receptor (GPCR) superfamily especially overexpressed in brain tumor cells [17, 18]. Following synthesis and characterization, the peptide was tagged on the surface of the nanoencapsulated DIM as a means of improving targeted therapy of high grade gliomas. Additionally, we outlined the proof-of-concept experiments *in vitro* and *in vivo* to explore the therapeutic potential of this novel nanopreparation against the debilitating effects of GBM.

## RESULTS

### *In silico* screening and characterization of SSTR2 peptides

**Figure 1:**
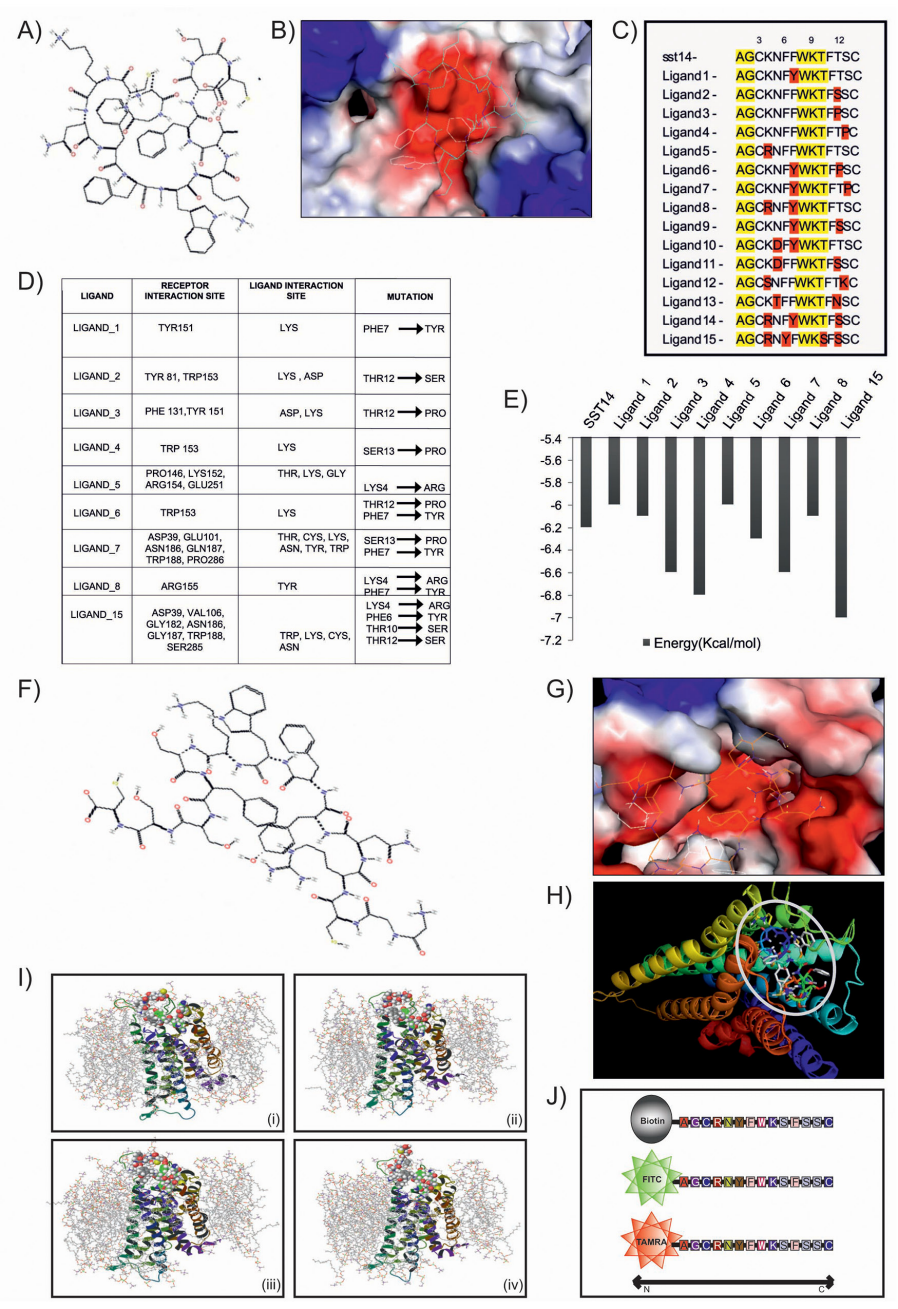
Design and *in silico* validation of potent peptide ligand for targeted delivery **(A)** & **(B)** Image represents chemical structure of SST14 and its binding with SSTR2. **(C)** & **(D)** Figure and table show mutated peptides and interaction of them with SSTR2. **(E)** Graph illustrates comparison of binding energy of various peptides with SST14. **(F)** & **(G)** Images represent chemical structure of ligand_15 and its efficient binding with SSTR2. **(H)** Patch dock result shows docking of ligand_15 (stick model) on SSTR2 (ribbon model). **(I)** Image represents molecular dynamic simulation of SSTR2- Ligand_15 complex at (i) 0 ns, (ii) 1.5 ns, (iii) 3.1 ns, and (iv) 4.8 ns. **(J)** Schematic diagram shows different types of tagging (biotin, FITC, TAMRA) at the N terminus region of synthesized SSTR2 peptide.

**Figure 2:**
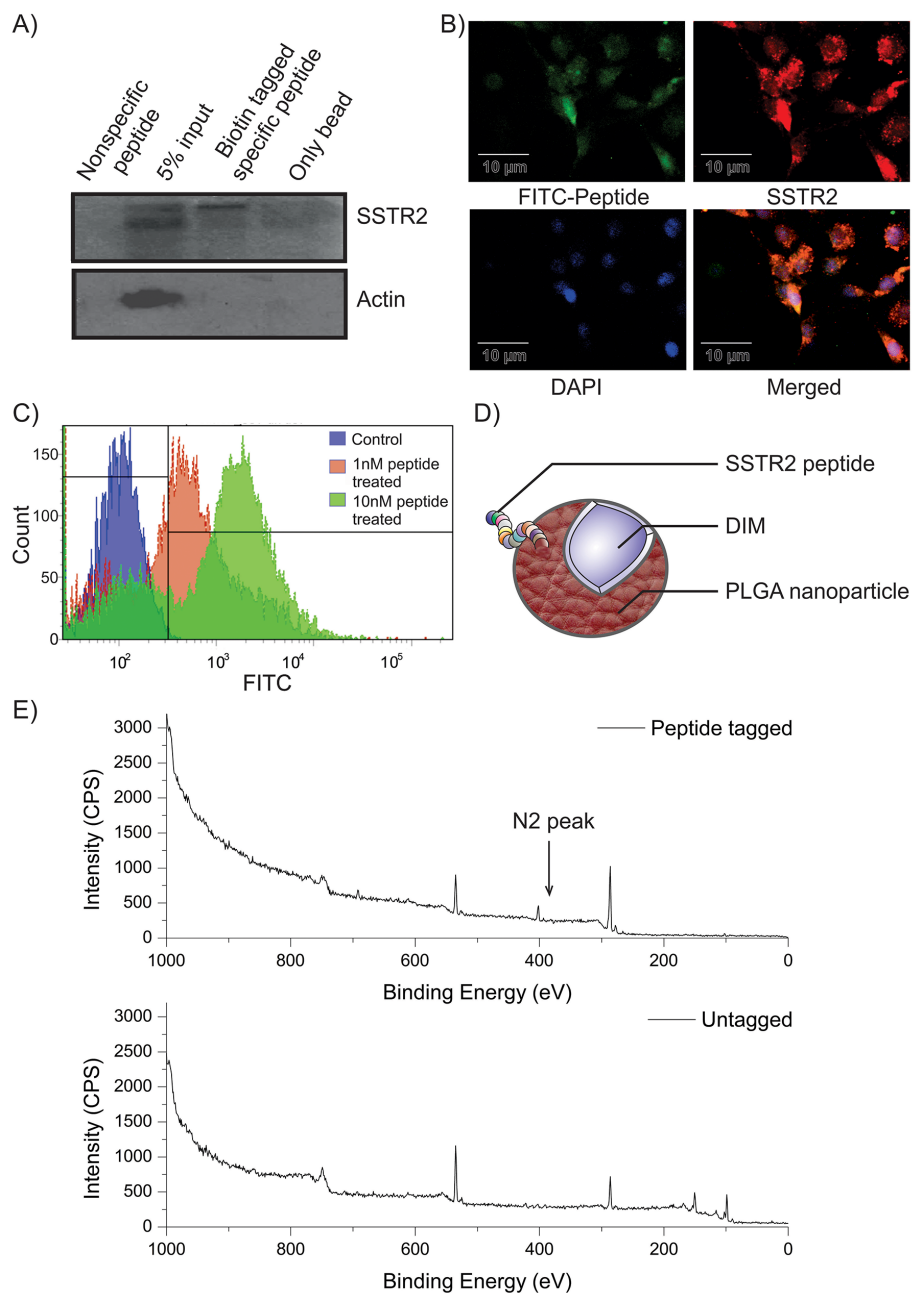
Characterization of SSTR2 peptide and confirmation of its tagging on nanoparticle surface **(A)** Specific band in lane-3 indicates pulled down SSTR2 by the biotin-tagged peptide. Actin was kept as the loading control. **(B)** Fluorescence microscopy confirms colocalization of both FITC-tagged peptide (green) and SSTR2 (red) in C6 cells. Cells with positive colocalization show yellow colour and nuclei have taken colour of DAPI (blue). **(C)** FACS data represents dose-dependent specific binding (shifting of peak) of FITC-tagged peptide treated C6 cells. **(D)** Presence of DIM within the polymeric nanoparticle and peptide on its surface are depicted in the cartoon diagram. **(E)** XPS spectrum of SSTR2 pep-DIM-NPs (Upper panel) and DIM-NPs(Lower panel) are illustrated. The peak at 535 eV, 286 eV and 402 eV represents Oxygen, -Carbon and -Nitrogen respectively. The peaks at 150 and 115 correspond to that of Silicon substrate.

Peptides against SSTR2 were designed by mutating its natural ligand somatostatin 14 or SST14 (Figure 1A–1B). Keeping the constant length as somatostatin, a library of 15 peptides was generated and mutation of each peptide was validated by using *Pymol* (Figure 1C). The mutations were created based on the hydrophobicity-hydrophilicity and polar-non polar nature of amino acids. Active peptides were screened on the basis of binding energy of ligand peptides and their interaction with SSTR2 (Figure 1D). *In silico* molecular docking study was undertaken to speculate the interaction and binding site of various designed peptides in SSTR2 model structure. It was observed that most of the ligands, viz., 3, 4, 6, 7 and 15 displayed lower binding energy and better binding affinity as compared to that of SST14 (Figure 1E). However, Ligand_15 (Figure 1F) was found to be most stable with binding energy -7.0 Kcal/mol. It showed better binding affinity and more interaction with SSTR2 (Figure 1G–1H), as it specifically interacts with SSTR2 at ASP^39^, VAL^106^, GLY^182^, ASN^186^, GLY^187^, TRP^188^ and SER^285^ by its LYS, ASN, TRP and CYS residues. The energy calculation of Ligand_15 represented the final energy expenditure for hydrogen bond, Vanderwaal and electrostatic interactions between the protein SSTR2 and the peptide (Supplementary Figure 1A). Furthermore, molecular dynamics membrane simulations were performed under the realistic biological condition where the SSTR2:ligand_15 complex was embedded under POPC (palmitoyloleoylphosphatidylcholine) membrane (a mimic of eukaryotic cell membrane) bi-layer system with 7604 number of solvent (SPC water) molecules (Figure 1I). Coordinates of the box of solute was a = 42.017209Å, b = 53.918604Å and c = 72.792316Å and boundary conditions of membrane system were orthorhombic having coordinate values of a = 62.017209Å, b = 73.918604 Å and c = 65.477 Å. The OPLS (Optimized Potentials for Liquid Simulations) force field was used throughout the equilibration at temperature 300K, isotropic pressure of 1.01325 Bar, isotropic and surface tension of 4000 dyne/cm. The closed conformation of the SSTR2 bound peptide at 3.1 ns indicated high stability and binding affinity even in the mimicry of biological conditions (Figure 1I, Supplementary Video 1). The RMSD graph for complex and ligand backbone showed good binding interaction and stability of peptide structure (Supplementary Figure 1B-1C). The Radius of gyration plot further supported very low fluctuation accounting for the stability of the complex (Supplementary Figure 1D). H-bond interaction between the receptor and peptide was shown in the H- bond plot analyzed by docking study (Supplementary Figure 1E). Based on the *in silico* data various *in vitro* analyses for the peptide (ligand_15) against SSTR2 were carried out. Peptides were procured from Thermo Fischer, and enzyme/fluorophore-tagged peptides (N-terminal tagging of Biotin, FITC and TAMRA) were also generated (Figure 1J). The purity of both untagged and tagged SSTR2 peptides was checked by Mass spectrophotometry and the results showed preferred peak at near 1642 mass to charge ratio with ∼100% intensity (Supplementary Figure 2). With the biotin tagged peptide, immunoprecipitation (IP) assay was performed which indicated specific binding of the peptide and SSTR2 proteins (Figure 2A, lane 3), whereas no bands were visible in case of whole cell lysates prepared from C6 (rat glioma) cells when incubated with scrambled non-specific peptide and only bead. The specific binding was further validated by immunofluorescence assay where colocalization of FITC tagged peptide and SSTR2 protein was noticed (Figure 2B). Furthermore, binding of SSTR2 receptor with different doses of the peptide was also quantified by FACS analysis (Figure 2C). Intensities of FITC were observed to be increased in SSTR2 expressing C6 cells treated with increased dose of peptide, indicating enhanced binding. TAMRA and FITC tagged scrambled peptides showed insignificant nonspecific binding to the cells in immunofluorescence assay (Supplementary Figure 3A) and FACS analysis (Supplementary Figure 3B) respectively, and the binding was not increased in dose dependent manner. It was proved further by cell viability assay and Immunoblotting (IB) that the specific SSTR2 peptide alone cannot alter the growth rate of C6 cells even upto 10 μM of dose (Supplementary Figure 3C), and also it has no ability to change the expression level of phospho ERK1/2, ERK1/2, phospho p38MAPK, p38MAPK and p21, which are downstream molecules of SSTR2 pathway (Supplementary Figure 3D). Therefore, the results further supported that the selected SSTR2 peptide, ligand_15, is an inert peptide.

### Preparation of PLGA encapsulated DIM and coupling of SSTR2 peptide with PLGA-DIM nanoparticles

The effect of the native form of DIM (Supplementary Figure 4A) was prominent in brain cancer cells, specifically in U87MG, C6 and DBTRG. The IC_50_ values of DIM for these three cell lines were 24, 28 and 20 μM respectively (Supplementary Figure 4B). But the expected Peaks 12 of DIM at around 247 ([M+H]^+^) or 269 ([M+Na]^+^) were missing in ESI mass spectrometric result (Supplementary Figure 4C), which suggests the absence of native DIM in rat brain. To facilitate the entry of DIM through BBB it was nanoencapsulated (nanotized) in PLGA by a modified emulsion-diffusion-evaporation method. After coupling by using EDC-NHS coupling method, the attachment of SSTR2 peptide to the PLGA-DIM nanoparticles (Figure 2D) was confirmed by ‘Nitrogen’ peak in XPS (Figure 2E, upper panel). The spectra obtained in the peptide tagged nanoformulation showed the peaks corresponding to Carbon (286 eV), Oxygen (535 eV) and most importantly, Nitrogen (401 eV). Nitrogen peak was absent in untagged PLGA-DIM nanoparticles (Figure 2E, lower panel). Nitrogen, a component of the peptide, present in the amide bond that formed between the nanoparticles and SSTR2 peptide via NHS activation. Therefore, the XPS-spectra confirmed the binding of the peptide on the surface of PLGA-DIM nanoparticles.

### Characterization of peptide tagged and untagged PLGA-DIM nanoparticles (SSTR2 pep-DIM-NP)

**Figure 3:**
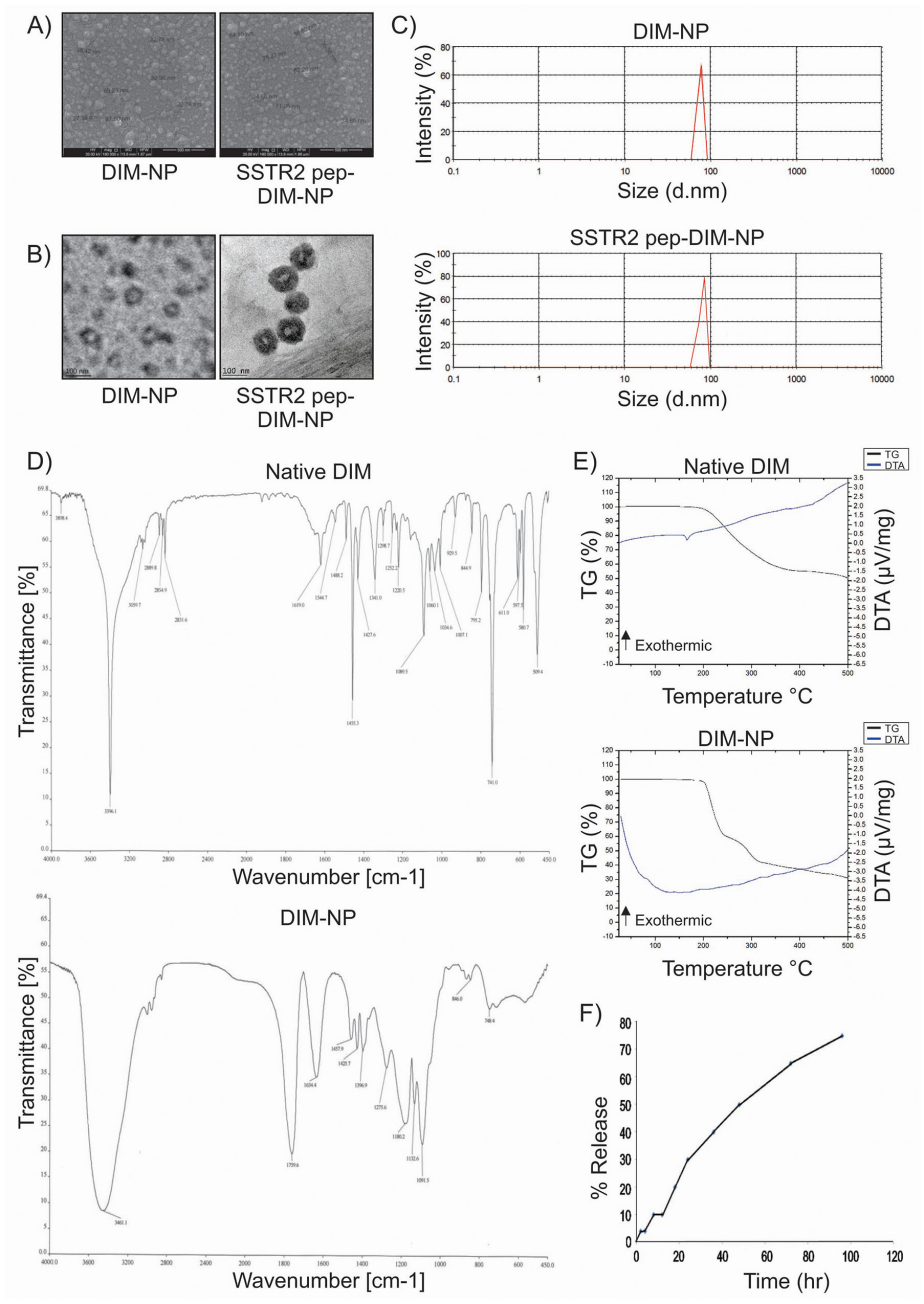
Characterization of DIM-NPs and SSTR2 pep-DIM-NPs **(A, B)** & **(C)** FE-SEM, TEM and DLS spectrum show spherical shapes and below 100 nm particle size. **(D)** FTIR spectra and **(E)** TG/DTA thermograms for native DIM and DIM-NPs and **(F)** kinetic release of DIM from PLGA nanoparticles.

The optimization study of untagged DIM-NP is displayed in Supplementary Table 1. Highest entrapment efficiency of the nanopreparation (75.20% ± 0.49%) was obtained at an optimum drug-polymer ratio of 1:5 (Supplementary Table 1). Scanning electron microscopy (SEM), Transmission electron microscopy (TEM) and Dynamic light scattering (DLS) analysis of the aqueous dispersion of both tagged and untagged nanopreparations showed that the NPs were more or less spherical in shape with size distribution in the range 27–87 nm for DIM-NPs and 28-98 nm for SSTR2 pep-DIM-NPs (Figure 3A–3C). Lyophilized powders of DIM-NP and SSTR2 pep-DIM-NP were found to be stable (Supplementary Table 2), readily dispersible in water, and could be stored at room temperature as well as at 4°C without any putrefaction or aggregation. The zeta potentials of DIM-NP and SSTR2 pep-DIM-NP were found to be 65±8.35 mV and 67±7.84 mV, suggesting high stability of the formulations. Fourier transform infrared spectroscopy (FTIR) was conducted to find out whether there was any structural change in the DIM-NPs as compared to native DIM (Figure 3D). The strong and wide peak at 3461.1 cm^-1^ correlated with the hydroxyl and amine groups overlapping each other in case of the nanoformulation. A similar peak of amine stretching was observed at 3396.1 cm^-1^ in case of native DIM. A C-H stretching was observed at 2,889 cm^-1^ in native DIM which was present in case of nanoencapsulated DIM also indicative of a possibility that nanoencapsulated DIM was equivalent to its native counterpart. The TG-DTA curves confirmed that DIM-NPs present thermal stability at a lower temperature range (up to 200 °C) (Figure 3E). DIM-NP was highly reactive due to its wide superficial area and, therefore, suffered quick thermal decomposition. The weight loss of the nanopreparation occured in two steps (230°C - 39% and 310°C - 16%), related to the exothermic events between the temperatures of 200°C-350°C (Figure 3E, lower panel). The TG-DTA curve of the nanopreparation did not display any specific peak relative to the melting point of pure DIM at 167°C reinforcing the fact that DIM was successfully encapsulated in the PLGA nanoparticles. Furthermore, release of DIM from both peptide tagged and untagged PLGA nanoparticles occurred in a controlled manner, with just 28% of the drug being released by 24 hr, and even up to 70% within 96 hr, suggesting that DIM was well-entrapped within the PLGA nanoparticles (Figure 3F).

### Internalization of SSTR2 peptide tagged NPs in glioma cells *in vitro*

**Figure 4:**
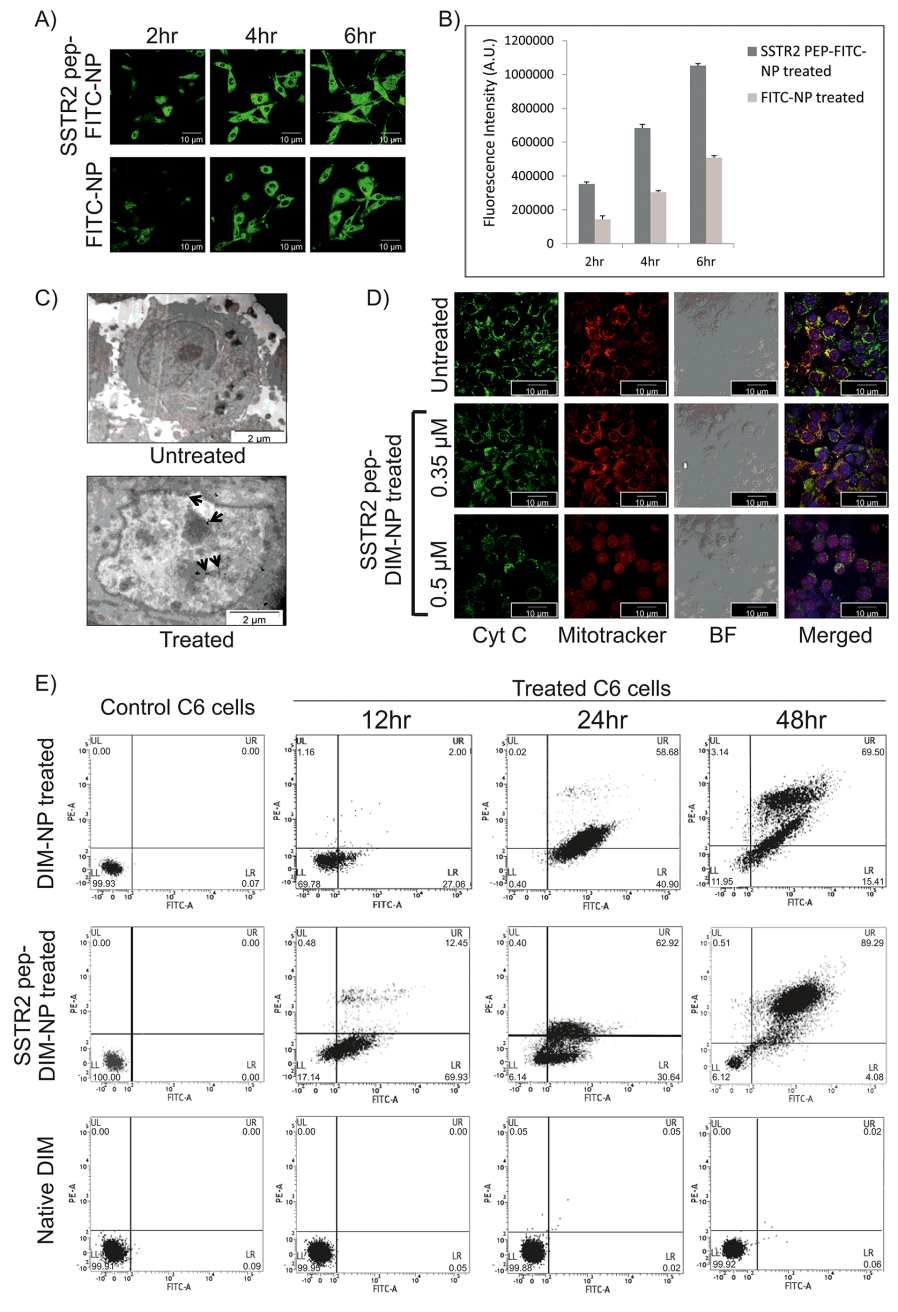
Celluler internalization and *in vitro* apoptotic effects of SSTR2 peptide-tagged nanoformulations **(A)** Image represents internalization of SSTR2pep-FITC-NPs and FITC-NPs in C6 cells after 2, 4 and 6 hrs of incubation. **(B)** Fluorescence intensity measurement of the images of Figure 4A shows higher intensity of SSTR2pep-FITC-NP treated group at each time point. **(C)** TEM image of SSTR2pep-DIM-NP treated C6 cells represents disrupted outer membrane integrity and destroyed cellular organelles, whereas untreated cells have intact morphology. Spots indicated by black arrows represent internalization of nanoencapsulated DIM inside the cells. **(D)** Fluorescence micrographs of untreated (Uppermost panel) and treated (Lower panels) C6 cells shows cytochrome C (Cyt C) release from mitochondria (stained with mitotracker red) after 12hr of SSTR2-pep-DIM-NP treatment. **(E)** FACS analysis evaluates apoptotic cell population after 12,24 and 48 hrs of treatment with 0.5μM of DIM-NP, SSTR2-pep-DIM-NP and native DIM. Left most panel shows control plot of untreated cells.

Cellular internalization of the nanoparticles takes place via endocytic pathway. Confocal laser scanning microscopy (CLSM) and TEM were therefore used to study internalization and intracellular trafficking of both SSTR2 peptide tagged and untagged PLGA nanoparticles in C6 glioma cells. As shown by CLSM studies in Figure 4A, robust fluorescent (green) signals in the plasma membrane and cytoplasm of the cells treated with SSTR2 pep-FITC-NP and FITC-NP over a span of 6hr was accounted for successful internalization of FITC in both cases. Initial docking (within 2hr) of SSTR2 pep-FITC-NPs on the plasma membrane was more in respect to untagged FITC-NPs. Furthermore, cellular binding and internalization of peptide tagged nanoparticles was found to be significantly higher than that of untagged nanoparticles (Figure 4A–4B), as fluorescence intensity was higher in SSTR2 pep-FITC-NP treated groups. In this case, FITC was used instead of DIM to visualize and validate the cellular internalization of particles released from the nanoencapsulation. These results were further corroborated by the observations made from TEM analysis which showed most of the nanoclusters were localized within the SSTR2 pep-DIM-NP treated cells (Figure 4C) that eventually underwent apoptosis, occurrence of which was completely absent in untreated control cells.

### Antiproliferative and apoptotic effects of SSTR2 pep-DIM-NPs on glioma cells

The effect of DIM, DIM-NP and SSTR2 pep-DIM-NP on cell viability was evaluated by MTT assay. Apparent growth suppressions on C6 glioma cells were observed upon DIM treatment depending on concentration, time and formulations. As shown in Supplementary Figure 5A, antiproliferative ability of the nanoformulations followed the order: SSTR2pep-DIM-NP>DIM-NP>DIM. DIM-NP seemed to be more effective than native DIM as the IC_50_ value after 48h treatment got reduced to 0.39 μM in case of C6 cells (Supplementary Figure 5A, lower left panel), whereas in case of native DIM the value was 28 μM (Supplementary Figure 5A, upper left panel). Significantly enhanced antiproliferative effect was achieved for SSTR2pep-DIM-NP displaying IC_50_ value at a much lower concentration of 0.3 μM (Supplementary Figure 5A, lower right panel). Furthermore, treatment with SSTR2 pep-DIM-NP at a concentration of 0.5 μM resulted in an enhanced antiproliferative effect. Therefore, we carried out further *in vitro* studies with the nanoformulations at 0.5 μM dosage. The next point to explore following antiproliferative studies was to estimate the apoptotic potential of the nanoformulations in C6 glioma cells. Initiation of apoptosis was observed within 12hr of SSTR2 pep-DIM-NP treatment, as microscopy revealed cytochrome C release from mitochondria, which was confirmed by the diffused fluorescence in higher dose (Figure 4D). Observations made from Flow-cytometric study revealed that the percentage of apoptotic cell death in C6 cells induced by SSTR2 pep-DIM-NP treatment (0.5 μm) for 24h and 48h was 62% and 89% respectively (Figure 4E). Treatment with DIM-NP at the same dosage and time periods yielded 58% and 69% of apoptotic cell death whereas no significant effect was observed with native DIM at this concentration. These results were further validated by scratch assay and colony formation assay. The scratch assay showed increased scratch diameter (Supplementary Figure 5B) after 48hr of treatment with SSTR2 pep-DIM-NP. The size of the C6 cell derived colonies (Supplementary Figure 5C) also got reduced upon SSTR2pep-DIM-NP treatment, indicating its anti-proliferative effects. Therefore, we believed that the enhanced antiproliferative and apoptotic effect of SSTR2 pep-DIM-NP was mainly owed to its elevated cellular binding facilitated by SSTR2 peptide and nanoformulation.

### Transport of SSTR2 pep-DIM-NP across the BBB *in vitro* and *in vivo*

**Figure 5:**
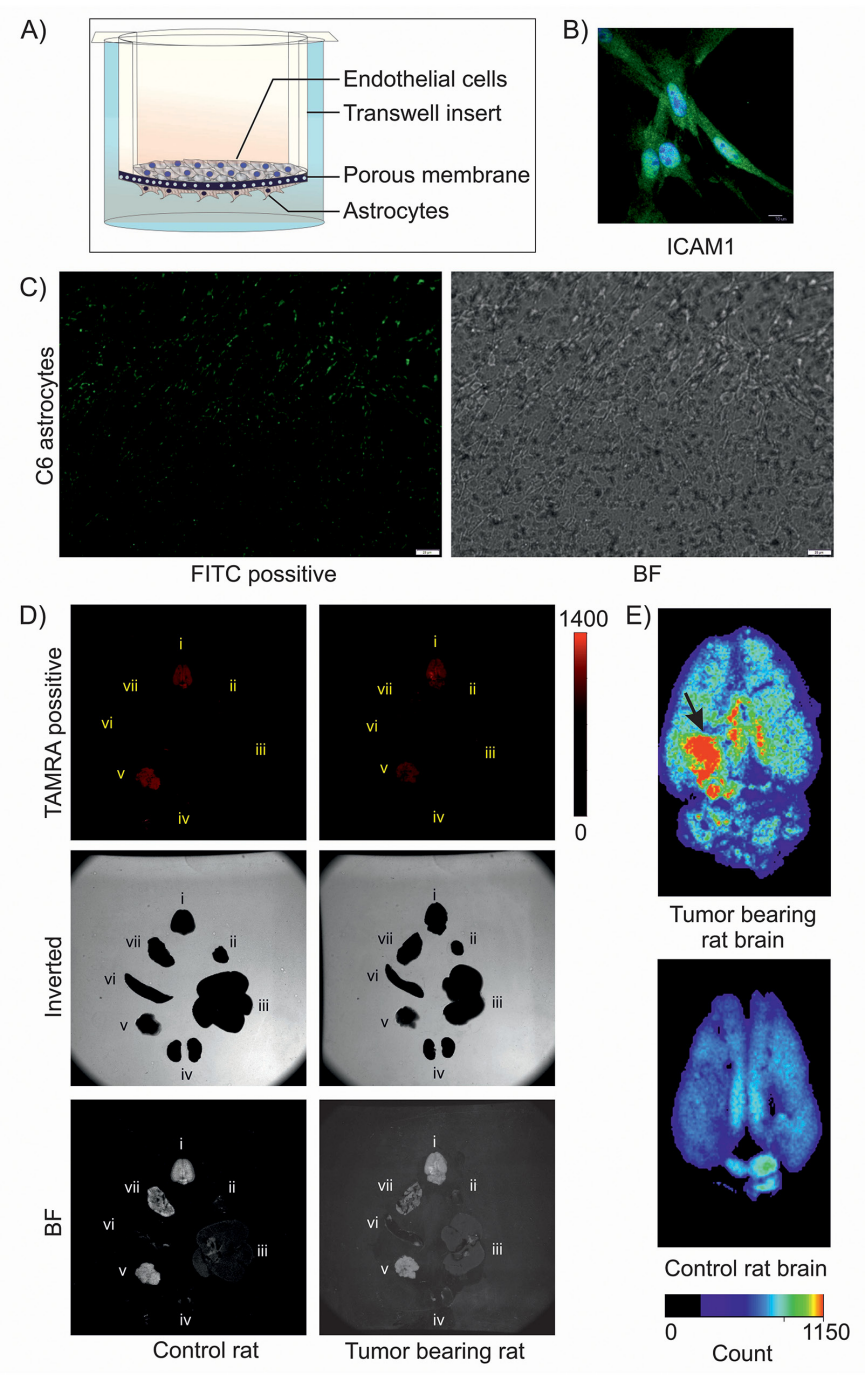
Transport of DIM nanoformulation through *in vitro* and *in vivo* BBB **(A)** Pictorial representation of *in vitro* BBB model. **(B)** ICAM1 staining of isolated primary rat brain endothelial cells. **(C)** Fluorescence imaging of C6 astrocytes shows binding of FITC-tagged SSTR2 pep-DIM-NPs passed through the tight junction of *in vitro* BBB. Bright field (BF) image shows C6 cells on the lower side of transwell insert. **(D)** Fluorescence (Upper), inverted (middle) and bright field (lower) imaging of multiple organs [brain (i), heart (ii), liver (iii), kidney (iv), Pancreas (v), spleen (vi) and lung (vii)] of control and tumor bearing rats represent binding of the TAMRA tagged-SSTR2 pep-DIM-NPs to the specific brain tumor region. **(E)** Enlarged images of rat brains shows enhanced and specific binding of nanoformulation to the tumor region (denoted by black arrow), which is absent in control rat brain (lower panel).

As we are aware of the fact that the blood brain barrier (BBB) renders majority of chemotherapeutics and targeted agents ineffective, we next sought to explore whether SSTR2 peptide tagged nanoparticles could cross the BBB efficiently. We therefore generated an *in vitro* BBB model which not only replicates the vascular properties but also mimics the dynamic responses of BBB and plays an important role in maintaining the brain homeostasis and neuronal functions. The *in vitro* BBB model was prepared by co-culturing endothelial cells and C6 astrocytes (Figure 5A), where the primary endothelial cells were identified by ICAM1 staining (Figure 5B), as ICAM1 is a well known marker for endothelial cells [19, 20]. TEER measurement revealed the values in the range of 200 to 350 Ω cm^2^, which indicated the formation of tight junction in the BBB model. Lead acetate used as positive control for TEER dependent *in vitro* BBB assessment (Supplementary Figure 6A). Nanoformulation caused decrease in TEER value, which indicates its passaging through *in vitro* BBB, whereas native DIM showed no effect on TEER value (Supplementary Figure 6A). From this data it is evident that native DIM cannot cross BBB. Passaging of FITC conjugated SSTR2 pep-DIM-NP was noticed by fluorescent microscopy, which also indicated towards the possibility of crossing of the drug through BBB *in vivo*. It has been noticed that the FITC conjugated peptide tagged nanoformulation not only crossed the *in vitro* tight junction, but also got associated to the C6 cells present on the lower surface of the transwell insert (Figure 5C). To further validate this phenomenon, TAMRA-conjugated SSTR2 pep-DIM nanoparticles were injected through the tail vein of SD rats and following two hours of drug induction, the animals were sacrificed and different body organs including brain were dissected out. The fluorescence imaging of the dissected body organs was done using an organ imager, which revealed high and specific binding of the formulation at the tumor region of rat brain (Figure 5D–5E). The observation clearly pointed out the ability of the peptide tagged nanoformulation to cross the BBB of the brain tumor bearing SD rats. Conversely, other body organs viz., heart, lung, liver, spleen, pancreas and kidney displayed negligible to almost no binding of fluorescent nanoparticles, which again emphasized the binding specificity of the peptide tagged nanoformulation towards tumor cells overexpressing membrane bound SSTR2 receptor. On the other hand, nonspecific peptide tagged nanoparticles were present in most of the body organs due to its nonspecific binding, but they were unable to bind specifically to the tumor region of rat brain (Supplementary Figure 6B). Furthermore, the presence of DIM in the brain tumor region of rats treated with SSTR2 pep-DIM-NP was observed by mass spectrometry (Supplementary Figure 7). Observations made from the study indicated towards the fact that the DIM nanoparticles not only targeted the tumor region successfully but also released the drug at the target destination in a sustained manner.

### Effect of SSTR2 pep-DIM-NP on intracranial tumor growth

**Figure 6:**
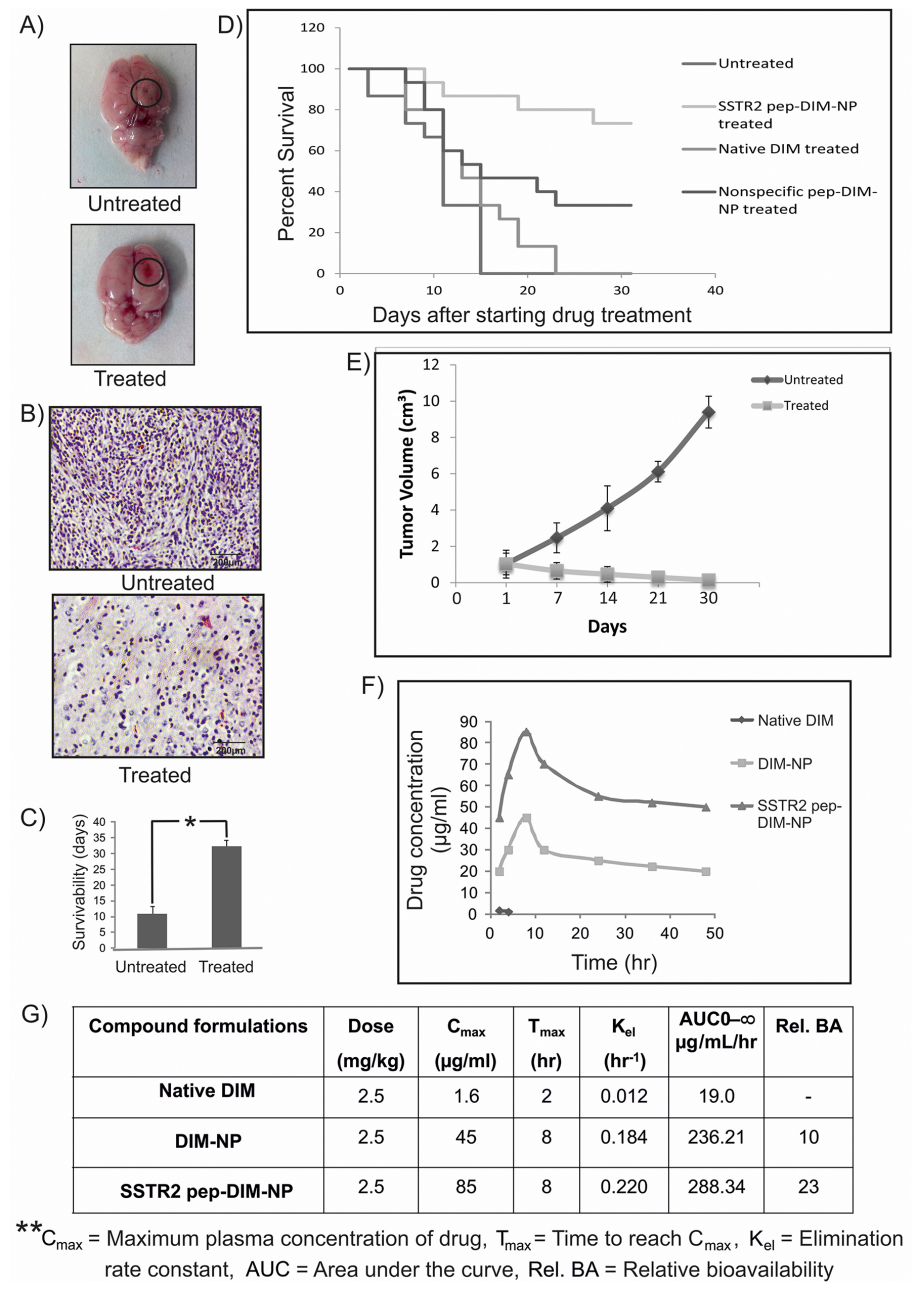
*In vivo* efficacy and bioavailability of SSTR2pep-DIM-NPs **(A)** Representative images of untreated (large) and treated (small) rat brain tumors, **(B)** H&E staining of their sections and **(C)** comparison of survivability between the two groups (n=6 in each group). Data is represented as mean ± SEM and *indicates P<0.001. **(D)** Figure represents overall survival curve for rats with orthotopic brain tumor. At the 31^st^ day after treatment maximum survival rate is 73.33% for SSTR2pep-DIM-NP treated group, whereas for untreated, native DIM treated and nonspecific peptide treated groups the percentages are 0%, 0% and 33.33% respectively (n=6 in each group). **(E)** Graphs showing the tumor volume as a function of time with and without SSTR2pep-DIM-NP treatment. **(F)** Graphical representation shows time-course of plasma concentrations over 48 hr following intravenous administration of 2.5 mg/kg native DIM, DIM-NPs and SSTR2pep-DIM-NPs to the adult SD rats (n=6 in each group). **(G)** Table represents the parameters of bioavailability studied for the nanoformulations.

The next issue that needed to be addressed was whether the BBB-penetrating peptide tagged PLGA nanoparticles were capable of inhibiting the growth of intracranial tumors. A nearly absolute growth inhibition and reduction in tumor volume (Figure 6A) was observed following 40 days long treatment with SSTR2 pep-DIM-NP whereas untreated group showed larger size of tumor. Observations made from histological analysis also suggested reduction in cellular density and development of spongy appearance in the treated tumor tissue (Figure 6B) which indicated towards the gradual death of the tumor cells. This high effectivity of the nanopreparation against tumor growth was further validated by the improved survival of SSTR2pep-DIM-NP treated tumor bearing rat groups which was increased almost 2-3 times than that of the untreated tumor bearing rat groups (Figure 6C). From the survival curve higher percentage of survival was noticed in SSTR2pep-DIM-NP treated group compared to untreated, native DIM treated and nonspecific pep-DIM-NP treated groups (Figure 6D). Tumors were generated by injecting C6 glioma cells in the flank region of the rats. Significant reduction in C6 tumor volumes in rat flanks were observed upon SSTR2 PEP-FITC-NP treatment, whereas in untreated groups the tumor volume increased considerably (Figure 6E, Supplementary Figure 8A–8B).

### SSTR2pep-DIM-NP is more bioavailable than native DIM

We further estimated the concentration of DIM that was released from the peptide tagged nanoformulation in the plasma of brain tumor bearing rat. Mean concentrations of DIM in rat plasma at different time points after intravenous administration (2.5mg/kg body weight for each) of SSTR2 pep-DIM-NP, DIM-NP and native DIM had been shown in Figure 6F. The profile of the concentration of untagged and peptide tagged DIM nanoparticles in blood plasma revealed that the relative bioavailability of them is 12 fold and 13 fold higher than that of native DIM respectively. The peak plasma concentration of DIM was obtained within 10 hr of drug administration. The table in Figure 6G showed significant increase in C_max_ (from 1.6 ng/ml for native DIM to 45 ng/ml and 52 ng/ml for DIM-NP and SSTR2 pep-DIM-NP respectively) and the AUC was found to be increased by ∼13 folds following treatment with peptide tagged nanoformulation in comparison to that of native DIM signifying improved bioavailability and better stability along with prolonged sustained blood circulation of SSTR2 pep-DIM-NP under physiological conditions. Moreover, histopathological studies conducted on different body organs of untreated and treated rats exhibited normal morphology thereby indicating nontoxic effects of our nanoformulation (Supplementary Figure 9).

### SSTR2 pep-DIM-NP induced EGFR pathway inhibition directed to apoptosis

**Figure 7:**
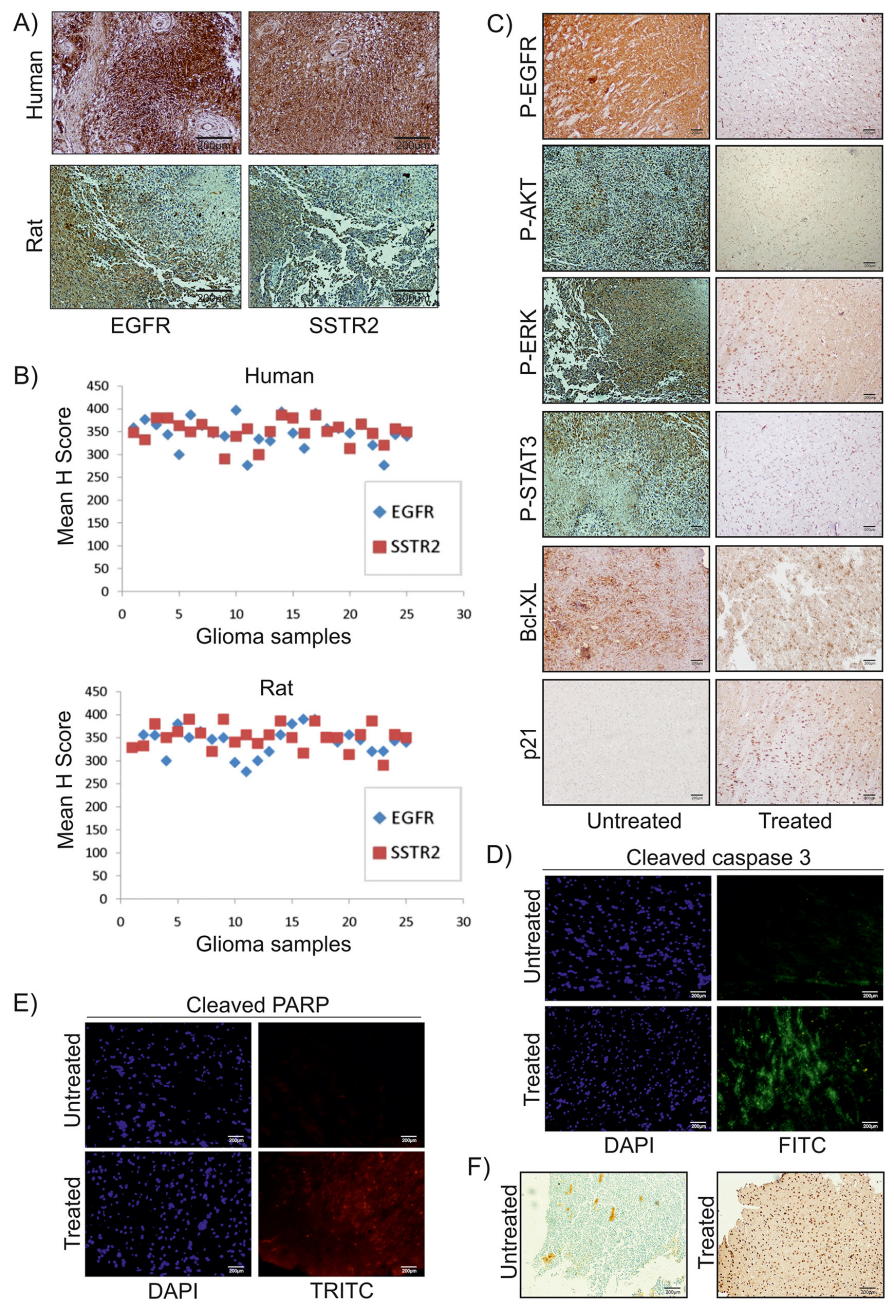
SSTR2pep-DIM-NP mediated inhibition of EGFR pathway *in vivo* Representative images of IHC analysis of **(A)** human and rat glioma samples (n=25 for each group) show EGFR and SSTR2 expression. **(B)** Scattered plot represents mean H-scores of EGFR and SSTR2 for both groups. Correlation coefficient (r) between mean H-scores are +0.2901 and +0.1504 for human and rat samples respectively. **(C)** IHC analysis for P-EGFR, P-AKT, P-ERK, P-STAT3, Bcl-XL, p21. **(D)** & **(E)** Immunofluorescence study for cleaved caspase 3 (FITC-green) and cleaved PARP (TRITC-red). **(F)** TUNEL positive cells in untreated and SSTR2pep-DIM-NP treated rat brain tumors. All images were captured in an upright microscope with 10X magnification.

**Figure 8:**
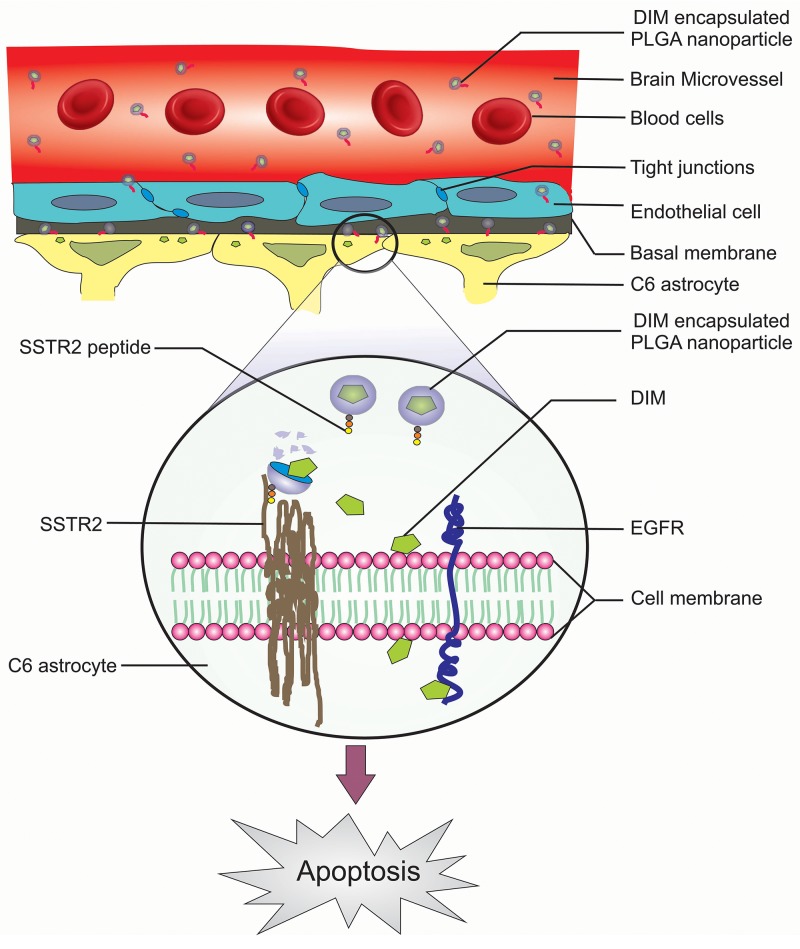
Schematic illustration of the strategy for SSTR2 peptide mediated targeted delivery of nanoencapsulated DIM through BBB of the rat Glioma model

Recent reports suggested that reduction in activation of EGFR pathway was linked to apoptosis in various cancer cells including glioma [21]. In this study, expressions of SSTR2 and EGFR were investigated in both human and rat glial tumor samples (n= 25 each) and all were found immunopositive (Figure 7A). Statistical analysis of immunohistochemical investigation of these samples clearly indicates a positive correlation between these two proteins (Figure 7B). Therefore, based on the findings made from the ongoing study it became essential to speculate whether the apoptotic effects of SSTR2 pep-DIM-NP were mediated via alterations in EGFR signalling pathway and its downstream members. IHC analysis suggested decreased activation of EGFR pathway members (viz. phospho EGFR, phospho-AKT, phospho-ERK and phospho-STAT3) was observed upon treatment of DIM nanoformulation (Figure 7C). Quantitative RT PCR data shows no change in mRNA expression of EGFR pathway members (Supplementary Figure 10A) upon SSTR2 pep-DIM-NP treatment, however decreased expression and activity of EGFR pathway members were consistent in both *in vitro* and *in vivo* condition (Supplementary Figure 10B). Moreover, the expression level of pro-survival protein Bcl-XL was also reduced; whereas expression of pro-apoptotic protein p21 was increased indicating induction of apoptosis (Figure 7C). These findings were further supported by the immunofluorescence analysis, where the expression levels of cleaved caspase 3 (Figure 7D) and cleaved PARP (Figure 7E) were found to be increased in tumor sections of animals treated with the nanoformulation when compared to the tumors of untreated groups. Cleavage of caspase 3 and PARP are known to be early apoptotic events followed by DNA strand break. The breakage of DNA strand in the treated tumor tissues was also confirmed by the increased number of TUNEL positive cells observed in tumor sections of rats treated with the peptide tagged nanoformulation (Figure 7F). These findings are further elaborated in the following schematic illustration displaying the strategy for SSTR2 peptide based targeted delivery of nanoencapsulated DIM through BBB in rat glioma model (Figure 8).

## DISCUSSION

Treatment of glioma, a primary malignant tumor of the brain, is one of the most challenging problems in healthcare due to the existence of obstacles like BBB that prevents entry of most of the chemotherapeutic agents [22]. To overcome the challenges associated with drug delivery, we developed a delivery system composed of brain-penetrating PLGA nanoparticles encapsulating DIM along with a novel synthetic peptide against SSTR2 receptor tagged on its surface. Extensive expression of SSTR2 has been previously reported in both low and high grades of glioma [18, 23], which makes it a susceptible target for peptide based drug delivery. In the present study, expression of SSTR2 was found to be very high in human and rat glioma samples (Figure 7A). Although SSTR2 has its specific endogenous ligand (somatostatin) but it is not much involved in tumor growth inhibition and therapy [24]. Computational studies suggested that out of several designed SSTR2 peptides, Ligand_15 could be used for targeted delivery of the DIM-NPs to the brain tumor region (Figure 1, Supplementary Figure 1). *In vitro* specificity of the selected peptide against SSTR2 was confirmed by immunoprecipitation, colocalization and flow cytometric studies (Figure 2A–2C). Moreover, the inertness of the peptide made it more useful as it only helped in drug targeting without any change in cell viability and downstream signalling pathway (Supplementary Figure 3C-3D). XPS analysis proved the efficient binding of SSTR2 peptide on the surface of DIM-NP, which in turn increased the target specificity of the nanoparticles (Figure 2E). Physico-chemical characterization studies revealed that the optimized PLGA nanoencapsulated DIM (Supplementary Table 1) were <100nm in size even after surface tagging with SSTR2 peptide (Figure 3A–3C), were readily dispersible in water, represented a higher stability (Supplementary Table 2) and that there was no structural change in DIM in its nanoform (Figure 3D). In addition, DIM experienced a sustained release from the positively charged nanoparticles (Figure 3F). Furthermore, it was observed that the SSTR2pep-DIM-NPs exhibited superior *in vitro* apoptotic effect than the DIM-NPs and native DIM (S5A) and this was attributed to its successful cellular internalization (Figure 4A–4C). A sophisticated *in vitro* BBB system was designed such that the vascular properties and the dynamic responses of BBB could be mimicked (Figure 5A–5B) [25]. SSTR2pep-DIM-NPs was found to not only cross the *in vitro* BBB system (Figure 5C), but also delivered the drug-load specifically to the SSTR2 overexpressed glioma tissue of the tumor bearing rats (Figure 5D–5E, Supplementary Figure 6). Antitumor efficacy study on tumor bearing rats showed significant tumor growth inhibition and better survivability (Figure 6A–6E), which occurred due to the improved bioavailability of nanoformulation (Figure 6F–6G). Other than SSTR2, EGFR was also highly expressed in glioma samples (Figure 7A–7B). EGFR signalling plays a significant role in the pathogenesis of glioma, and therefore, inhibition of EGFR-mediated oncogenic signalling leads to apoptosis [21]. In the present study, results from the IHC and IB analysis suggested that upon sustained release from the nanoformulation, DIM inhibited EGFR pathway activation in both SSTR2 and EGFR overexpressed rat glioma samples (Figure 7A–7C, Supplementary Figure 10). Moreover, decreased expression of Bcl-XL and increased expression of p21, cleaved caspase 3 and cleaved PARP along with occurrences of TUNEL positive apoptotic cells (Figure 7C–7F) amplified the apoptotic role of SSTR2pep-DIM-NP. These findings therefore, confirmed that SSTR2 peptide mediated targeted delivery of DIM nanoparticles through BBB led to apoptotic death of glioma cells through inhibition of the EGFR pathway.

In summary, this nanoformulation suggests a potential therapeutic strategy for glioma, which can efficiently cross the BBB and exert apoptotic and antitumorigenic effects.. Therefore, we anticipate that brain penetrating target based nanoformulation will have significant clinical impact in the near future.

## MATERIALS AND METHODS

### Cell culture and reagents

Rat glioblastoma (C6) and human glioblastoma (DBTRG-05 MG and U87MG) cell lines were obtained from ATCC (American Type Culture Collection) and NCCS, Pune. These cells were cultured in DMEM supplemented with 10% fetal bovine serum and 1% penicillin/streptomycin in 5% CO2 at 37°C. Cells were treated with 3,3’-diindolylmethane (DIM), Nanoencapsulated DIM (DIM-NP) and SSTR2 peptide tagged nanoencapsulated DIM (SSTR2-pep-DIM-NP) as required.

### Animal maintenance

Adult SD rats (180–220 gm or ∼100 gm; 49 days or 21 days old) were received from the animal house of Indian Institute of Chemical Biology. The animals were maintained at laboratory conditions (12:12, dark:light cycle) fed with standard pellet diet and water supplied with ad libitum. All experiments were performed in accordance to the CPCSEA guidelines with the approval of the animal ethics committee of CSIR-Indian institute of Chemical Biology.

### Cell viability assay

Cell viability assay was performed using MTT [3-(4,5-dimethylthiazol-2-yl)-2,5-diphenyl-tetrazolium bromide]. C6 cells cultured in 96-well dishes and treated with native DIM, empty nanoparticle, DIM-NP and SSTR2-pep-DIM-NP for 24 and 48 hrs were subjected to MTT assay by following established protocol [26]. Other than C6, cell lines like DBTRG-05 MG, U87MG were also cultured in 96-well dishes and treated with native DIM for 48 hr, which further subjected to MTT assay. MTT assay values of the spectrophotometric absorbance of only SSTR2 peptide (ligand_15) treated C6 were also measured after 24 and 48 hr. All readings were taken by ULTRA Multifunctional Microplate Reader at 550 nm.

### Preparation of nanoencapsulated DIM

A modified emulsion-diffusion-evaporation method was used to formulate the nanotized DIM. In brief, 500 mg of PLGA (50:50, MW: 7,000–17,000) was dissolved in 5 ml of ethyl acetate, and 50 mg of the commercially available pure DIM was added to this mixture. It was kept at room temperature for 30 min with intermittent vortexing. The organic solution was then emulsified with an aqueous phase containing didodecyldimethylammonium bromide (DMAB). The resulting oil-in-water emulsion was stirred at room temperature for 3 hr before homogenizing at 15,000 rpm for 5 minutes with a high-speed homogenizer (Polytron PT4000; Polytron Kinematica, Lucerne, Switzerland). Subsequently, the organic solvent was removed by rotary evaporation and the aqueous phase containing DIM was sonicated for 30 minutes. The resulting aqueous emulsion was centrifuged at 35,000 rpm for 1 hr and the nanoprecipitate was suspended in phosphate buffered saline (PBS), along with 20% sucrose and glucose (used as a cryoprotectant), lyophilized and stored at room temperature for future use. The lyophilized preparation of nanotized DIM was found to be devoid of ethyl acetate and DMAB. The method for the preparation of DIM nanoparticles was optimized on the basis of particle size, zeta potential and entrapment efficiency with respect to varied drug-polymer ratio as well as at different concentrations of the surfactant (DMAB) used.

### Peptide mutation

The peptides against somatostatin were designed by mutation keeping the length constant as somatostatin using *Pymol* an open-source, user-sponsored, molecular visualization system created by Warren Lyford DeLano and commercialized by DeLano Scientific LLC. The three amino acids (Tryptophan, Lysine, Threonine) (except Ligand_15 in which Threonine was mutated with Serine) at beta-turn were kept conserved to maintain the integrity of structure. The other residues were mutated based on hydrophobicity/-hydrophilicity and polar, non polar nature of residue as each amino acid has a one or more specific amino acid for substitution.

### *In silico* analysis

Energy minimization, virtual screening and SSTR2 binding study was performed as follows:

(i) After mutation, the peptides were energy minimized using Avogadro with MMFF94 force field and steepest descent algorithm results in more stable conformation for virtual screening and docking study.

(ii) The Model structure of SSTR2 was downloaded from uniprot P30874. Loops and missed portion of the structure were built, modeled using Modeller software and analyzed using Protein portal model server. All the designed 15 mutant peptides in a library were virtually screened using Autodock Vina against the model SSTR2 protein including the natural somatostatin peptide SST14. The active peptides were selected on the basis of their binding energy and interaction. Molecular docking using Autodock 4.2 was performed to explore the probable binding site of each peptide with SSTR2. The interaction sites and stability of the selected peptides with respect to somatostatin was represented. Nonspecific peptide with amino acid sequence “AGCANFFWKTFTAA” was also designed which was not able to dock with SSTR2.

(iii) The molecular dynamics membrane simulation represents SSTR2-Ligand_15 (protein-peptide) complex embedded in POPC membrane bilayer system with 7604 number of solvent (SPC water) molecules. It was built with the help of Desmond membrane builder. Size of the box of solute was 42.017209A, 53.918604A and 72.792316A and the boundary conditions of the membrane system were orthorhombic with coordinate values of 62.017209, 73.918604 and 65.477. The Optimized Potentials for Liquid Simulations (OPLS) force field was used throughout the equilibration at 300.0K, isotropic pressure of 1.01325 Bar and surface tension of 4000. It was run for 50 ps with 1.2 ps interval for NPT and NVT equilibration. After two stage equilibrium for 50 ps each, the system was put under molecular dynamics simulation [by Desmond Molecular Dynamics System, Copyright (c) 2004-2012 D. E. Shaw Research. Copyright(c) Schrodinger, LLC] for 4.8 ns under same environmental condition in OPLS-AA force field.

### Synthesis of SSTR2 peptide and coupling with PLGA-DIM nanoparticles

Peptide (ligand_15) and nonspecific peptide were chemically synthesised and tagged at its N-terminal, with either Biotin, FITC or TAMRA. The mass of the SSTR2 peptide was checked by mass spectrometry. For targeting glioma, DIM nanoparticles were tagged with SSTR2 peptide by EDC-NHS coupling. Briefly, 100 ml of PLGA-coated DIM nanoparticle suspension was activated by 200 ml of 400 mM 1-Ethyl-3-[3-dimethlyaminopropyl] carbodiimide hydrochloride (EDC or EDAC) and 200 ml of 100 mM N-hydroxysulfosuccinimide (NHS). The mixture was gently shaken for one hour at room temperature. To this, SSTR2 peptide was added and kept under gentle shaking for 4 hr. The nanoparticles were collected by centrifugation at 6000 rpm for 15 min and washed twice with Sterile Milli-Q water. The resultant nanoparticles were suspended in 1 ml nuclease free water and used for further analyses. Same procedure was repeated for the nonspecific peptide.

### Flow cytometric analysis

C6 cells were treated with 0.5μM of untagged DIM-NP, SSTR2-pep-DIM-NP and native DIM for 12, 24, 48 hrs, followed by staining with Annexin V/Propidium iodide (PI) and analyzed using a flow cytometer as stated previously [27]. Cell surface binding of FITC conjugated peptide[procured from Thermo Fisher Scientific Inc. (USA)] was also analyzed by Flow cytometry. SSTR2 peptide and nonspecific peptides (1 or 10 nM) were incubated with a suspension of 10^6^ C6 cells for 2 hr followed by washing with PBS. Data were acquired using FACS Calibur and analysed by CellQuest software (Becton Dickinson, Franklin Lakes, NJ).

### Immunoprecipitation (IP) assay

Cells were incubated for 24 hr in complete media supplemented with 50 μM biotin tagged SSTR2 peptide or biotin tagged scrambled peptide (synthesized from Thermo Fischer Scientific, USA). After washing thrice in PBS, whole cell lysates were prepared using lysis buffer (50 mM Tris: pH 7.4, 500 mM NaCl 0.4% SDS, 5 mM EDTA, 1 mM DTT, and complete protease inhibitor (Roche) and sonicated as required. Triton X-100 (2% final) was added to the lysates and centrifuged at 16,000xg. Supernatants were precleared with 10 μl streptavidin bead (1 mg/ml) for 30 min at 4°C. Supernatants were collected and incubated with 600 μl Dynabeads (MyOne Steptavadin C1; Invitrogen) overnight at 4°C. Beads were collected and washed five times for 15 min at room temperature in lysis buffer containing 0.1% deoxycholate before examined by Western blot analysis.

### Immunoblotting (IB)

After 48 hr treatment of SSTR2 peptide (1 and 10 pM/nM/μM), C6 cells were harvested in lysis buffer and whole cell lysates were prepared using standard protocol. After nanoformulation treatment whole cell lysates were prepared from rat glioma tissues and C6 cells of treated and untreated batches by standard procedures. 70 μg of cellular proteins were separated on SDS-PAGE and subjected to IB using primary antibodies against Phospho-EGFR (Y1068), EGFR, AKT, Phospho-AKT (S473), STAT3, Phospho-STAT3 (Y705), ERK1/2, Phospho-ERK1/2(Y202/T204), Phospho-p38 MAPK, p38 MAPK, p21 and Actin (Cell Signaling Technology and Santa Cruz Biotechnology). Actin was used as loading controls. Bands were detected using the Amersham ECL detection system (GE Healthcare).

### Quantitative real time PCR (qRT-PCR)

Total RNA was extracted from tissue and cells using TRIzol reagent (Invitrogen, NY, USA) according to the manufacturer’s instructions. For each sample, 2 μg of RNA was converted to cDNA using RevertAid First Strand cDNA Synthesis Kit (Thermo Scientific, Glen Burnie, Maryland, USA). The samples were subsequently used for qRT-PCR analysis using Power SYBR Green Master Mix on 7500 Fast real time PCR system (Applied Biosystems, Foster City, CA, USA). RPL19 served as internal controls (normalization).

List below are the primers (rat) used for qRT-PCR analysis:

RPL19 : F-5′-ATCGCCAATGCCAACTCT-3′

R-5′-GAGAATCCGCTTGTTTTTGAA-3′

EGFR : F-5′-TCTGCACAGGCTGGGTGAAA-3′

R-5′-GGGTGGGTCTCCTGGTTAGC-3′

AKT : F-5′-GAAGGCCACAGGTCGCTACT-3′

R-5′-CGGTCGTGGGTCTGGAATGA-3′

ERK : F-5′-TAACCAGCCCAGCACACCAA-3′

R-5′-CAAGCATGGGAGAGGCCTGA-3′

STAT3 : F-5′-TCACACGCCACTCTGGTGTT-3′

R-5′-AGGAGGCGAGACTCTTCCCA-3′.

### Physical characterization of DIM nanoformulations

**(i) Particle size measurements** - The particle size of the dispersion was measured with Nanosizer 90 ZS (Malvern Instruments, Southborough, MA). Measured size was presented as the average value of 20 runs, with triplicate measurements within each run. The sample was prepared by taking 1 mg of the lyophilized DIM-NP and SSTR2-pep-DIM-NP powder in 10 ml of distilled water and the particle size was accurately estimated by dynamic light scattering measurements. Zeta potential of the synthesized nanoparticles was measured by Zetasizer Nano ZS (Malvern Instruments, Southborough, MA, USA). Prior to analysis, the solutions were filtered through a 2 μm filter. Each sample was measured in triplicate.

**(ii) Scanning electron microscopy (SEM)** - Morphological characteristics of the newly synthesized DIM-NP and SSTR2-pep-DIM-NP were observed by scanning electron microscope model FEI Quanta 250 (USA). A minute quantity of sample is directly placed to aluminium stub with double side adhesive carbon tape, followed by gold coating before observation by SEM.

**(iii) Transmission electron microscopy (TEM)** - The size and morphology of DIM nanoformulations were examined using a JEOL JEM-2100 transmission electron microscope (JEOL, Inc., Peabody, MA, USA) at an acceleration voltage of 300 kV. One drop of nanoparticle suspension was dispersed in DI water, lyophilized and mounted on a thin film of amorphous carbon deposited on a copper grid (300 meshes). After drying under clean conditions the grid was examined directly with the transmission electron microscope.

**(iv) Entrapment efficiency and *in vitro* release study** - Centrifugation method was performed to determine the encapsulation efficiency (EE). The particles were precipitated. A certain proportion of fresh particles was dissolved in ethyl acetate. The entrapment efficiency was measured by using a UV-VIS spectrometer model Shimadzu (Singapore). The drug was detected at 280 nm. The EE parameter was calculated as follows:

$$EE=W_{t}-W_{d}/W_{t}$$

Where W_t_ and W_d_ describes the total DIM added and the extracted DIM into the supernatant, respectively. The EE calculated from the above formula was 75%.

*In vitro* release profiles of DIM from PLGA 50 nanoparticles were determined in phosphate buffer saline (PBS; pH 7.4). The release of DIM from PLGA 50 nanoparticles was measured till 96 hrs.

**(v) FTIR study of the synthesized DIM nanoparticles** - The effects of encapsulation process on the chemical group and the interaction between the components was studied by performing a fourier transform infrared spectroscopy (FTIR) model Spectrum TWO (Perkin Elmer, USA). The FTIR spectra ranging between 500 cm^-1^ and 4000 cm^-1^ were obtained by mixing samples with KBr powder (Infrared grade).

**(vi) Storage stability of DIM nanoparticles** - Physical stability of the nanopreparation during prolonged storage in room temperature (25±2°C) as well as at 4°C was determined routinely by monitoring changes in zeta potential, particle size, and drug content. External parameters such as temperature and light are of principal importance for long-term stability of any formulation. A high zeta potential of the nanopreparation aids in dispersion to remain physically stable.

**(vii) Simultaneous TG-DTA** - The thermal stability of the samples was confirmed by simultaneous TG and DTA. The curves were obtained in the temperature range from 25 to 500°C, using aluminium crucibles with about 5 mg of samples, under dynamic air atmosphere (100mL/min) and heating rate of 20°C/min.

### Cytochrome c release assay

C6 cells were seeded on coverslips and treated with SSTR2 pep-DIM-NP for 12 hr and the release of cytochrome c from mitochondria was noticed by the established procedure. Cells were observed under Olympus BX61 fluorescence microscope at 60X magnification. Images were captured and processed using Image-Pro Plus software (Media Cybernetics, Silver Spring, MD, USA).

### Soft-agar colony formation assay and two-dimensional scratch (wound healing) *assays*

In 0.35% agarose complete medium, 5,000 cells were mixed and plated on the bottom layer of 0.7% agarose–complete medium in 35 mm plates. The culture media containing 500nM SSTR2 pep-DIM-NP was changed every alternative day during the 14 days of cell growth. Colonies were counted in three different microscopic fields of Olympus IX81 microscope and also captured by using Image Pro Plus imaging software. Each experiment was performed at least thrice in triplicates.

For scratch assay, equal number of cells were seeded on 35 mm plates and allowed to reach confluency. Scratches were made by a 200 μl pipette tip and after 48 hr SSTR2 pep-DIM-NP treatment migration of cells was observed at premarked positions. Untreated scratched plates were kept as control.

### Orthotopic brain tumor model

Sprague–Dawley rats (200–250 g) were anesthetized and subjected to intracranial injection with early passaged C6 cells (1×10^6^). The entire experiment was done according to the methods described in earlier reports [28]. After 10 or 40 days, the tumor bearing rats were sacrificed using a thionembutal overdose and tumors were collected from the brains for further study. Portions of tumors were also fixed in 10% buffered formalin and embedded in paraffin for histology and immunohistochemical (IHC) analysis.

### *In vitro* BBB model

Primary cultures of rat brain capillary endothelial cells (RBEC) were prepared from 3-week-old rats using selective media. This culture method has been adapted from previously described techniques [29]. When the cultures reached ∼80% confluency, the endothelial cells were passaged by a brief trypsinization and used to construct *in vitro* model of BBB. Subsequently C6 astrocytes were seeded on bottom side of the collagen-coated polyester membrane of the transwell inserts. After the overnight adhesion of C6 cells, endothelial cells were seeded onto the upper side of the inserts and placed in the wells of a 12-well culture plate. From Day 1, the culture medium was supplemented with 500 nM hydrocortisone. The *in vitro* model of BBB was developed within 3 days. Trans-endothelial electrical resistance (TEER) was also measured with the help of Epithelial Voltohmmeter (*WPI* EVOM^2^) to confirm the functionality of the tight-junctions. TEER values were measured and plotted in a bar graph after 10^-8^ M, 10^-6^ M and 10^-4^ M of lead acetate (positive control) treatment and 10^-9^ M, 10^-8^ M and 10^-7^ M of native DIM and Pep-DIM-NP treatment. 10 nM of Fluorescence peptide tagged DIM nanoparticles (FITC conjugated SSTR2-pep-DIM-NP) were added to the upper chamber of this BBB model and the passaging of particles was noticed by fluorescence microscopy of C6 astrocytes of the lower chamber after 12 hrs.

### Drug plasma concentration measurement

Adult SD rats were injected with 2.5 mg/kg body weight of free DIM, DIM-NP or SSTR2-pep-DIM-NP as suspension in water. After cardiac exsanguinations blood samples were collected in EDTA coated tubes at 2, 4, 8, 12, 24, 36 and 48 hrs. Plasma from all samples was separated by centrifugation at 10,000 g for 5 minutes and subjected to further processing and HPLC analysis as described in earlier reports [26]. The concentrations were quantified by calculating the area under peaks by using UV detector at 254 nm.

### Mass spectrometry

Native DIM treated, SSTR2 pep-DIM-NP treated and untreated rat brain tumor tissues were frozen in liquid nitrogen and then crushed using a cooled mortar and pestle. 200 μl of diethyl ether was added to the crushed tissue and bath sonicated for 5 min. After vortexing the samples were subjected to centrifugation for 10,000 rpm for 10 minutes and the supernatants were transferred to a new 1.5 ml microcentrifuge tube for electrospray ionization (ESI) mass spectrometric analysis and kept for evaporation. Finally, 5 μl of the concentrated extract was used for mass spectrometry.

### Histology & immunohistochemistry (IHC)

Normal and tumor tissues of rat and human brain and tissues of other body parts were embedded in paraffin after 10% neutral buffered formalin fixation. Sections (5 μm) were prepared from paraffin-embedded blocks and used for histology and IHC analysis. For histological analysis, standard hematoxylin and eosin (H&E) staining protocol was used to stain the tissue sections. In case of IHC, antigen retrieval of tissue sections with citrate buffer was performed followed by blocking with serum for 30 min as discussed previously [30]. The sections were incubated with primary antibodies against SSTR2, EGFR, Phospho-EGFR, Phospho-ERK1/2, Phospho-AKT, Bcl-XL, p21 (Abcam & Cell Signaling Technology) overnight at 4°C. Finally DAB stained sections were visualized under light microscope Olympus BX61 [31].

### TUNEL assay

Identification of inter nucleosomal DNA strand breaks, a characteristic feature of apoptosis, was done by TUNEL assay. It was performed on SSTR2 pep-DIM-NP treated and untreated rat brain tumor tissues (paraffin sections). TdTFragEL DNA fragmentation detection kit (Calbiochem, Oncogene Research Products) was used to detect apoptosis, according to manufacturer’s instructions.

### Fluorescence and confocal microscopy, *in vitro* TEM and immunofluorescence study

C6 cells were subjected to immunofluorescence studies after the treatment of FITC tagged peptide for 24 hr. Cells were fixed, permeabilized and blocked with BSA before incubation with SSTR2 primary antibody (Abcam) overnight at 4°C, followed by TRITC tagged secondary antibodies for 1 hr at room temperature under dark condition [32]. To check the internalization of FITC encapsulated PLGA nanoparticles (FITC-NP) (0.5 μM) and SSTR2 peptide tagged FITC encapsulated PLGA nanoparticles (SSTR2 pep-FITC-NP) (0.5 μM) in C6 cells confocal microscopy was used after 2, 4 and 6 hrs of incubation. ImageJ software was used for fluorescent intensity measurement. C6 cells treated with SSTR2 pep-DIM-NPs (for 24 hr) were pre-fixed with 2.5% glutaraldehyde and 2% paraformaldehyde solution, post-fixed with 1% osmium tetroxide, dehydrated with a series of alcohols and infiltrated with resin. The resin sample block was trimmed, thin-sectioned and collected on copper grids. Before examining under the TEM, these grids were stained by uranyl acetate and lead citrate and air-dried. Tissue immunofluorescence of tumor sections was conducted for cleaved caspase 3 and cleaved PARP (Cell signalling technology) using the established protocol [33]. Images were captured using DP71 camera in a BX-61 microscope and analyzed using Image Pro Plus software.

### Biodistribution of nanoparticles

TAMRA conjugated SSTR2 peptide coupled nanoencapsulated DIM (200 μl of 1 mg/ml) were injected in tumor bearing and control rats through tail vein. Nonspecific peptide coupled nanoformulation was also injected in tumor bearing rats. After 3 hr tissues from seven different organs: kidney, spleen, liver, brain, heart, lung and pancreas were collected for imaging using Kodak Image Station 4000 MM PRO. For visual illustrations of fluorescence signals, color maps were generated using Carestream MI SE 5.0.2.30.

## XPS

For compositional information of the surface of PLGA nanoparticles and to prove the binding of SSTR2 peptide on PLGA surface, X-ray Photoelectron Spectroscopy (XPS) analysis was carried out using an XPS facility (VSW Ltd., UK) attached in a UHV (base pressure in the XPS chamber was 5×10^-10^ mbar) compatible nanocluster deposition unit [34]. In this experiment, MgKa x-ray, having an energy of 1253 eV (or 9.89 Å) was used and the photoelectrons produced were analyzed by a hemispherical electron analyser of a mean radius of 150 mm.

### Statistical analysis and densitometry

All statistical calculations were done using GraphPad (QuickCalcs, http://www.graphpad.com/quickcalcs/index.cfm) calculator and H-test was calculated manually. Student’s t-test was used for analysis of statistical significance.

## SUPPLEMENTARY MATERIALS FIGURES, TABLES AND VIDEO





## Abbreviations

DIM: 3,3’-diindolylmethane
GBM: Glioblastoma multiforme
BBB: Blood brain barrier
EGFR: Epidermal growth factor receptor
SSTR2: Somatostatin receptor 2
PLGA: Poly (lactic-co-glycolic acid)
GPCR: G protein-coupled receptor
I3C: Indole-3-Carbinol
POPC: Palmitoyloleoylphosphatidylcholine
RMSD: Root-mean-square deviation
TG/DTA: Thermogravimetric/differential thermal analysis
EDC: (1-ethyl-3-(3-dimethylaminopropyl) carbodiimide hydrochloride)
NHS: N-Hydroxysuccinimide
XPS: X-ray photoelectron spectroscopy
DIM-NP: DIM encapsulated PLGA nanoparticle
SSTR2 pep-DIM-NP: SSTR2 peptide tagged PLGA nanoparticles having DIM in its core
FITC: Fluorescein isothiocyanate
TAMRA: Tetramethylrhodamine
TEER: Transendothelial electrical resistance
Akt: Protein kinase B
ERK: Extracellular signal–regulated kinases
MAPK: Mitogen-activated protein kinases
STAT3: Signal transducer and activator of transcription 3
ICAM-1: Intercellular adhesion molecule-1
Bcl-XL: B-cell lymphoma-extra large
PARP: Poly (ADP-ribose) polymerase
C_max_: Maximum concentration
AUC: Area under the curve
SD rats: Sprague Dawley rats
TUNEL: Terminal deoxynucleotidyltransferasedUTP nick end labelling
