# Supplementary material for: Anti-SSTR2 peptide based targeted delivery of potent PLGA encapsulated 3,3’-diindolylmethane nanoparticles through blood brain barrier prevents glioma progression
Source: Oncotarget. 2017 Jun 27;8(39):65339–58. doi: 10.18632/oncotarget.18689 (PMC5630335; doi:10.18632/oncotarget.18689)
Supplement: Supplementary file 1 [file oncotarget-08-65339-s001.pdf]

Anti-SSTR2 peptide based targeted delivery of potent PLGA encapsulated 3,3'-diindolylmethane nanoparticles through blood brain barrier prevents glioma progression

SUPPLEMENTARY MATERIALS

A)

Ligand 15	Final Intermolecular Energy	= -5.00 kcal/mol
	vdW + Hbond + desolv Energy	= -5.41 kcal/mol
	Electrostatic Energy	= +0.41 kcal/mol
	Final Total Internal Energy	= -7.89 kcal/mol
	Torsional Free Energy	= +11.34 kcal/mol
	Unbound System's Energy	= -7.89 kcal

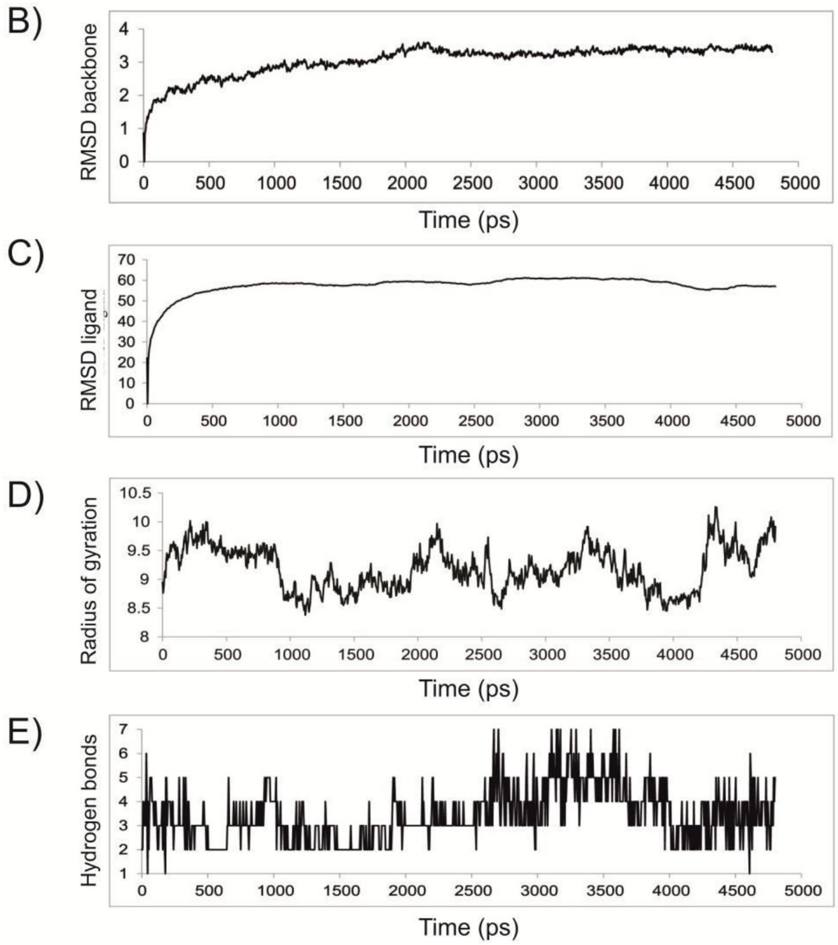

Supplementary Figure 1: Energy calculation of molecular docking and study of different molecular dynamics membrane simulation parameters. **(A)** Table shows final energy calculation results for SSTR2 peptide (ligand_15). Vdw = Vanderwaals force and H bond = Hydrogen bond. Graphs represent root mean square deviation (RMSD) plot of **(B)** receptor backbone and **(C)** ligand. **(D)** and **(E)** are the graphical representations of changes in radius of gyration and hydrogen bond formation.

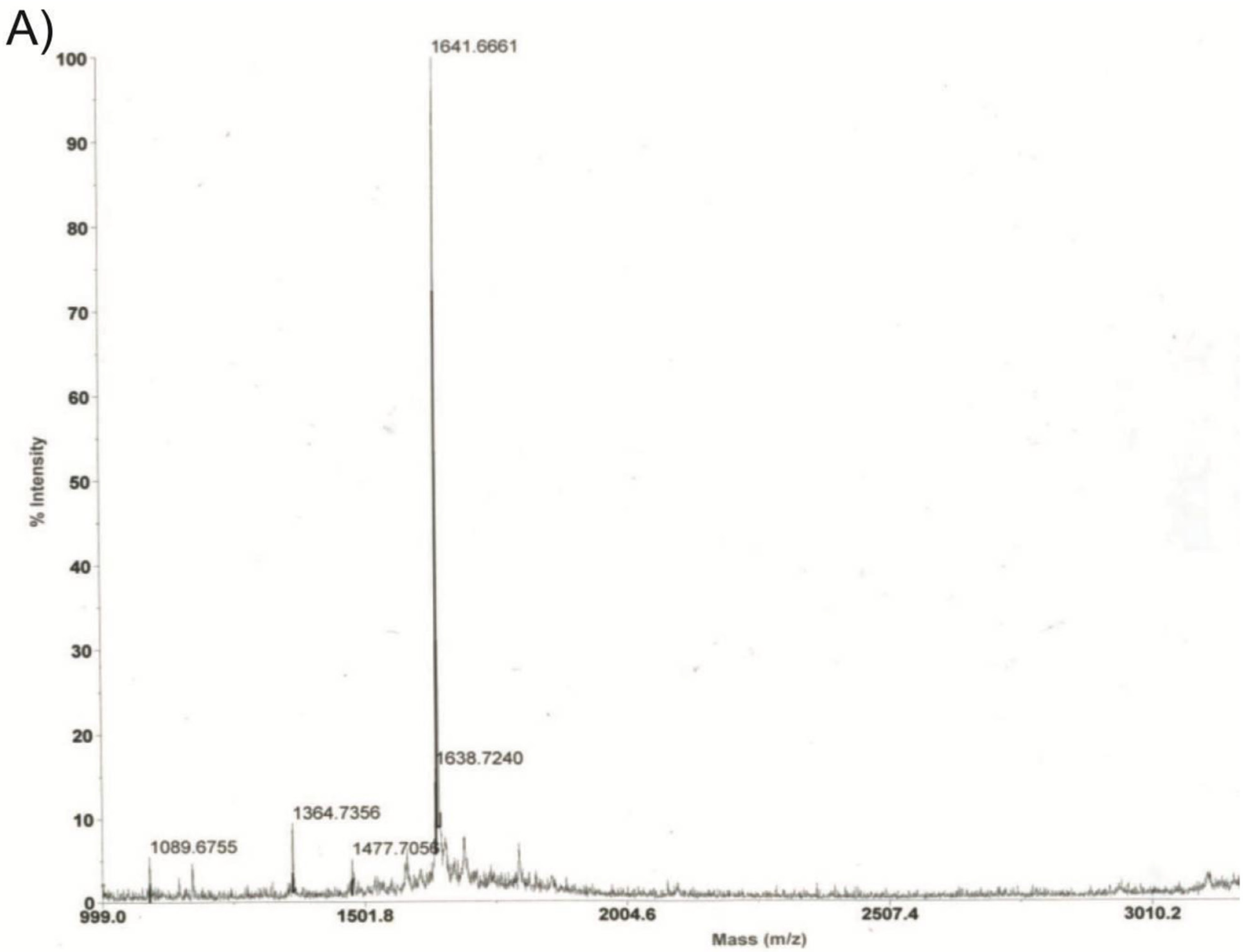

Supplementary Figure 2: Mass spectrometric data representing molecular mass of SSTR2 peptide (ligand_15).

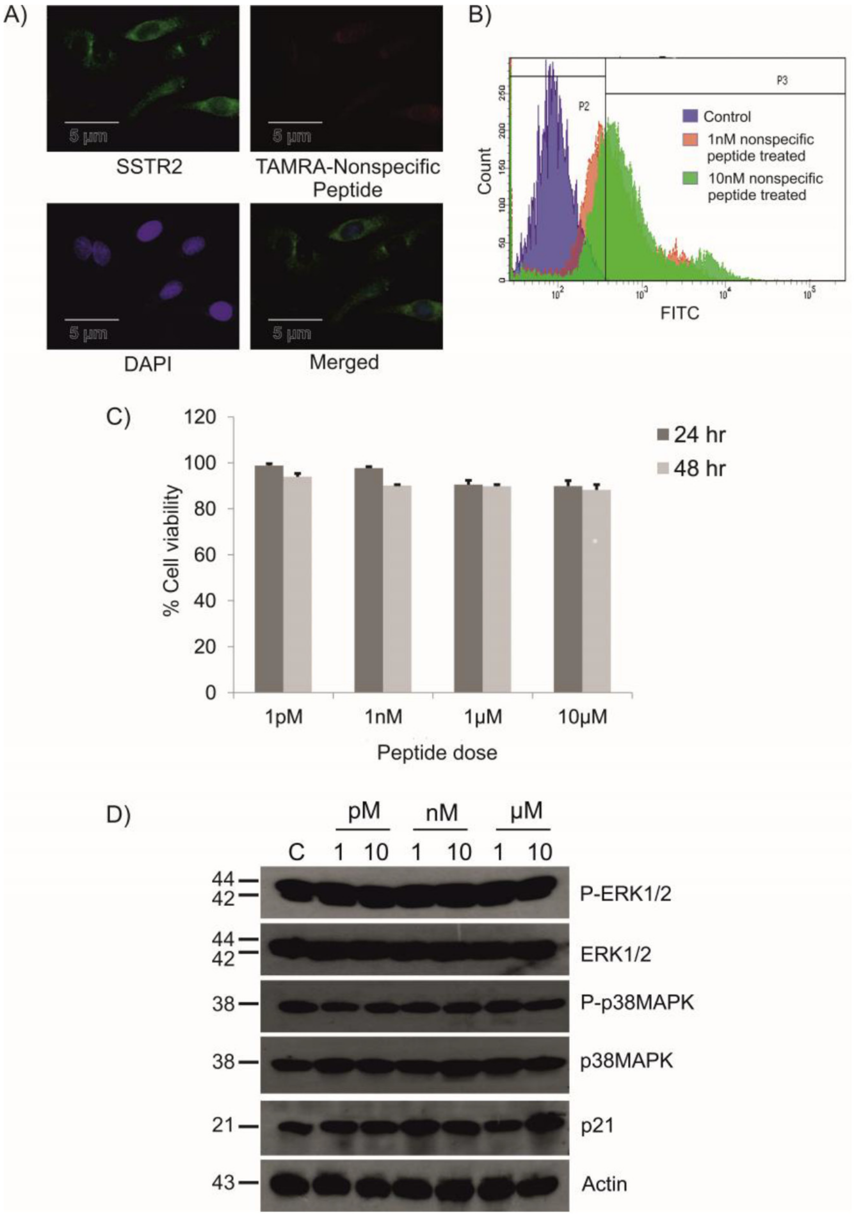

Supplementary Figure 3: Nonspecific peptide binding assessment and *in vitro* efficacy study of SSTR2 peptide. **(A)** Fluorescence microscopy shows very low binding of TAMRA tagged nonspecific peptide to cells. It represents no colocalization of nonspecific peptide (red) and SSTR2 (green) in C6 cells. **(B)** FACS data represents no significant dose-dependent increase in binding (shifting of peak) of FITC-tagged nonspecific peptide to C6 cells. **(C)** Graph represents percentage of viable cells after 24 and 48 hrs treatment of 1pM, 1nM, 1µM and 10 µM SSTR2 peptide treatments. **(D)** Expressions of P-ERK1/2, ERK1/2, P-p38MAPK, p38MAPK, p21 and actin upon treatment of 1 and 10 picomolar (pM), nanomolar (nM) and micromolar (µM) of SSTR2 peptide.

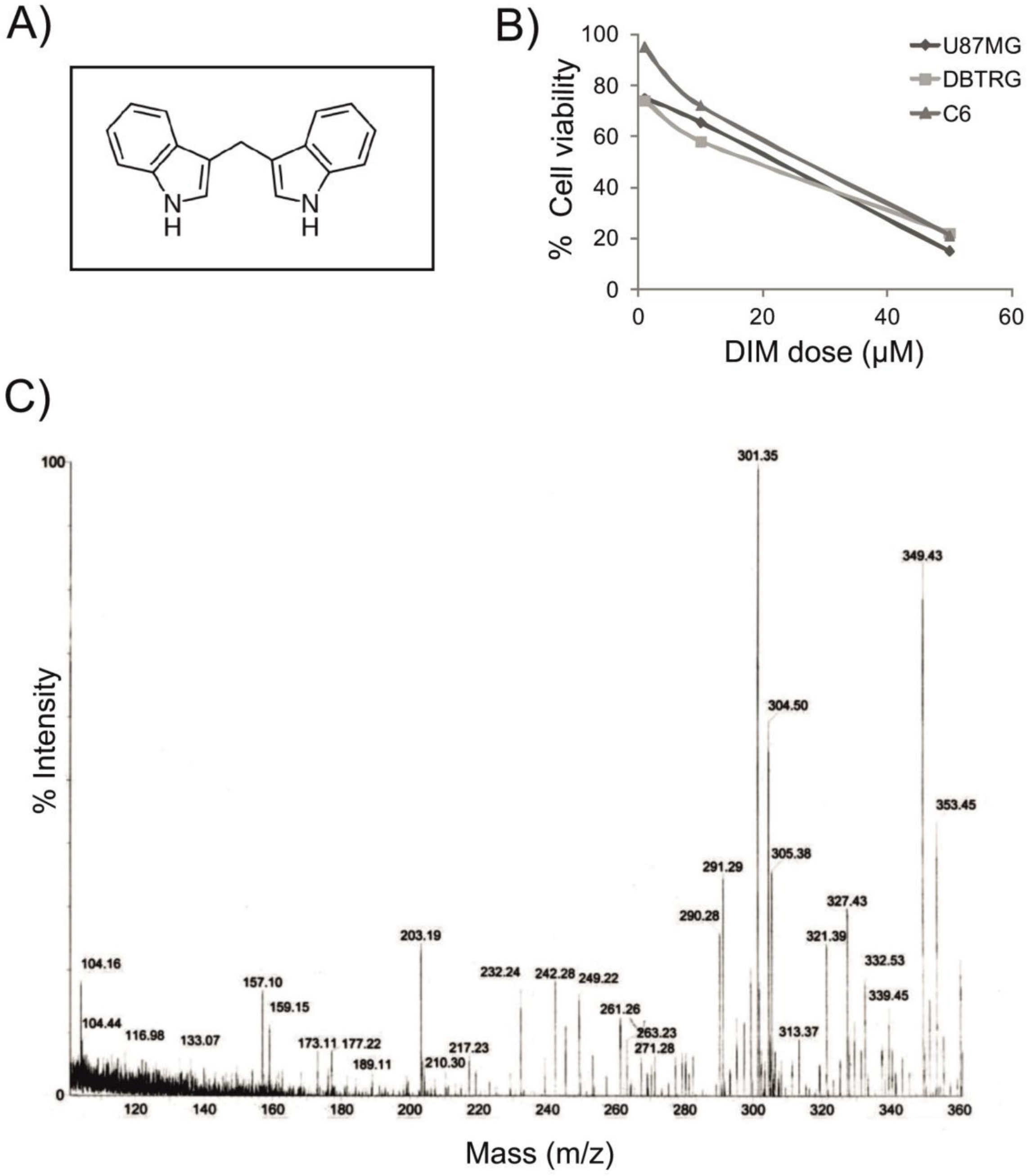

Supplementary Figure 4: Determination of drawbacks of native 3,3' diindolylmethane (DIM). (A) Chemical structure of DIM. **(B)** MTT data represents % cell viability of U87MG, DBTRG and C6 cell lines. IC_{50} values of them are 24, 28 and 20 μM respectively. **(C)** ESI-mass spectrometry shows no specific peak of DIM in case of native DIM treated rat brain tissue extract.

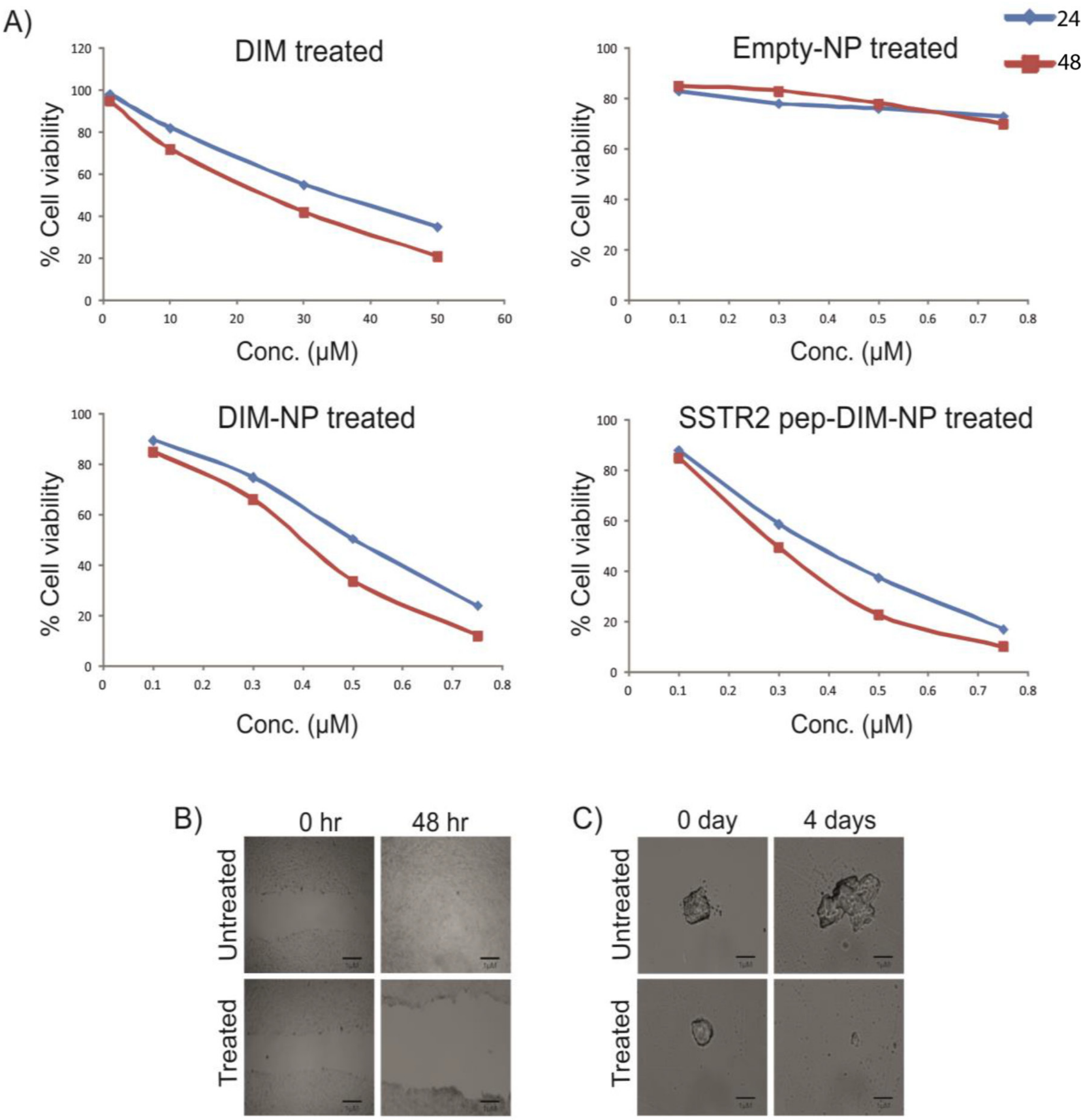

Supplementary Figure 5: Effect of nanoformulations on cell viability, migration and colony formation. **(A)** Figure represents % cell viability after 24 and 48 hrs treatments of Native DIM, Empty NP, DIM-NP and SSTR2 pep-DIM-NP to C6 cells. **(B)** Panels show representative images of untreated and SSTR2pep-DIM-NP treated C6 cells. Scratches on cell monolayer treated for 48 hr shows the increased wound gap at 500 nM whereas decreased gap can be observed in untreated cells. **(C)** Figure represents increased colony size in untreated C6 cell groups after 4 days. SSTR2pep-DIM-NP treated cells show reduced colony size at the same time point.

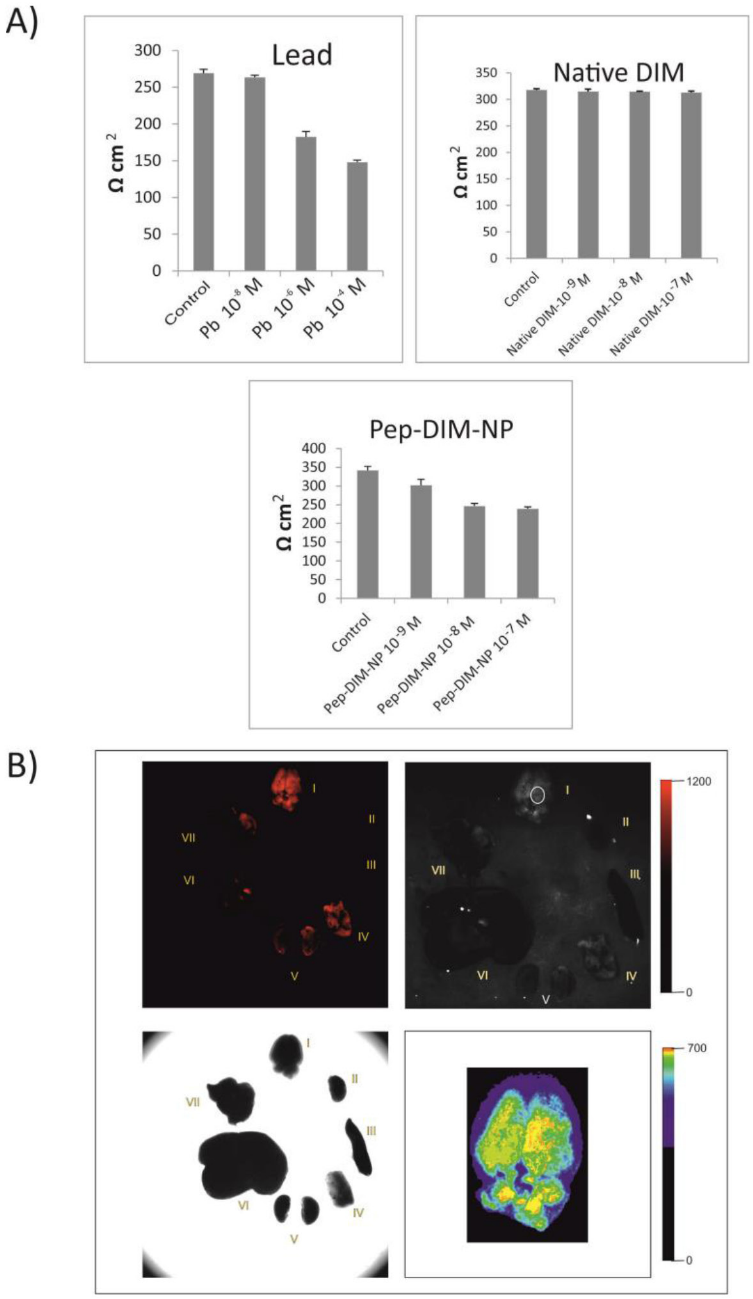

Supplementary Figure 6: *In vitro* testing of BBB functionality and *in vivo* nonspecific peptide binding analysis. **(A)** Comparison of TEER value (Ωcm^2) of lead acetate, native DIM and SSTR2 pep-DIM-NP treated *in vitro* BBB models. **(B)** Fluorescence (Upper left), inverted (lower left), bright field (upper right) and enlarged brain (lower right) imaging represent no specific binding of the TAMRA tagged-nonspecific peptide-DIM-NPs to the brain tumor region (denoted by white circle in bright field image). Multiple organs [brain (i), heart (ii), spleen (iii), pancreas (iv), kidney (v), liver (vi) and lung (vii)] have taken from the treated tumor bearing rat.

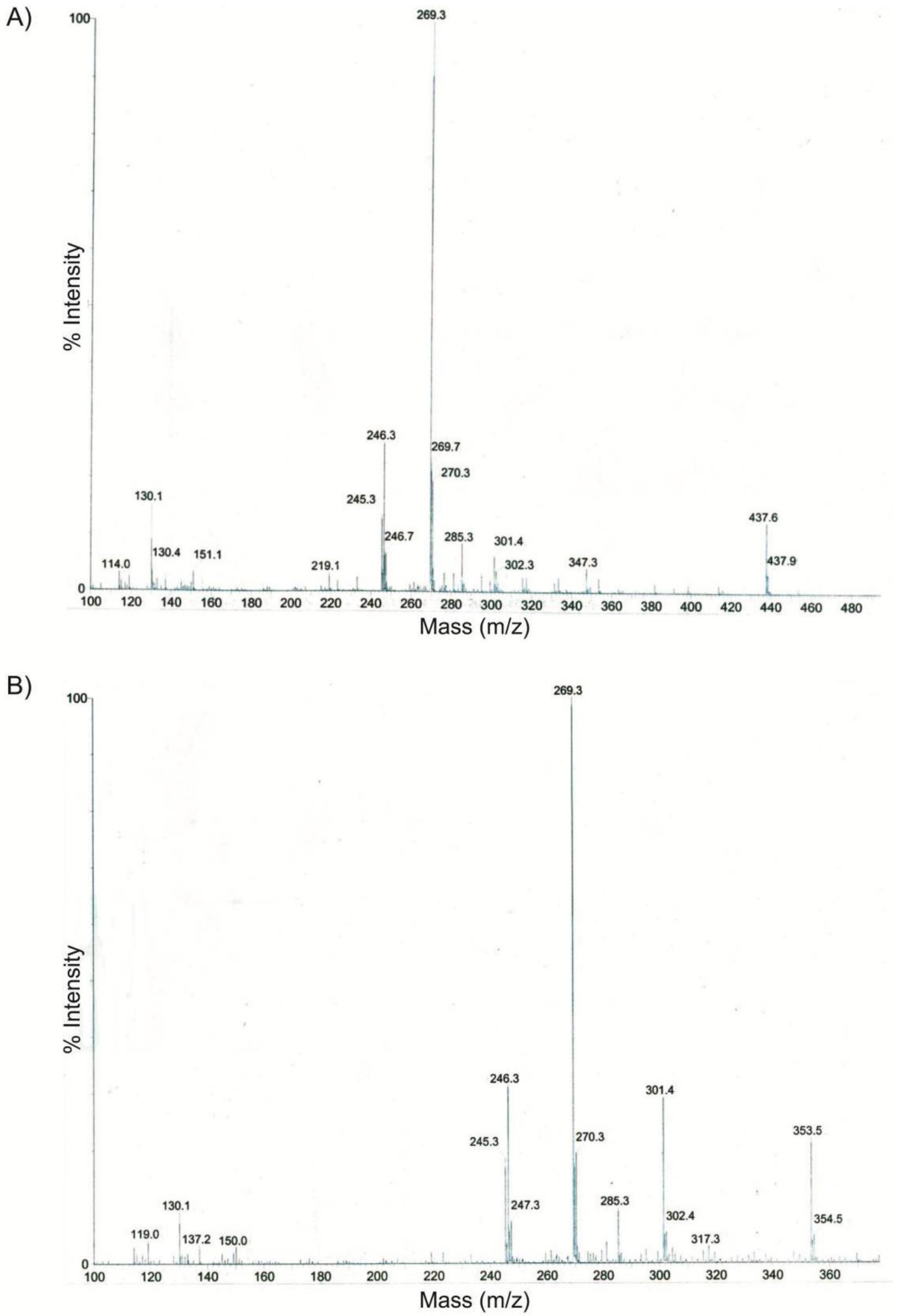

Supplementary Figure 7: ***In vivo*** **internalization study of DIM in rat brain tumors. (A)** Highest intensity peak in graphical representation denotes molecular mass of native DIM. **(B)** Graph shows same peak with highest intensity defining exact molecular mass of DIM in tissue extracts of nanoformulation treated rat brain.

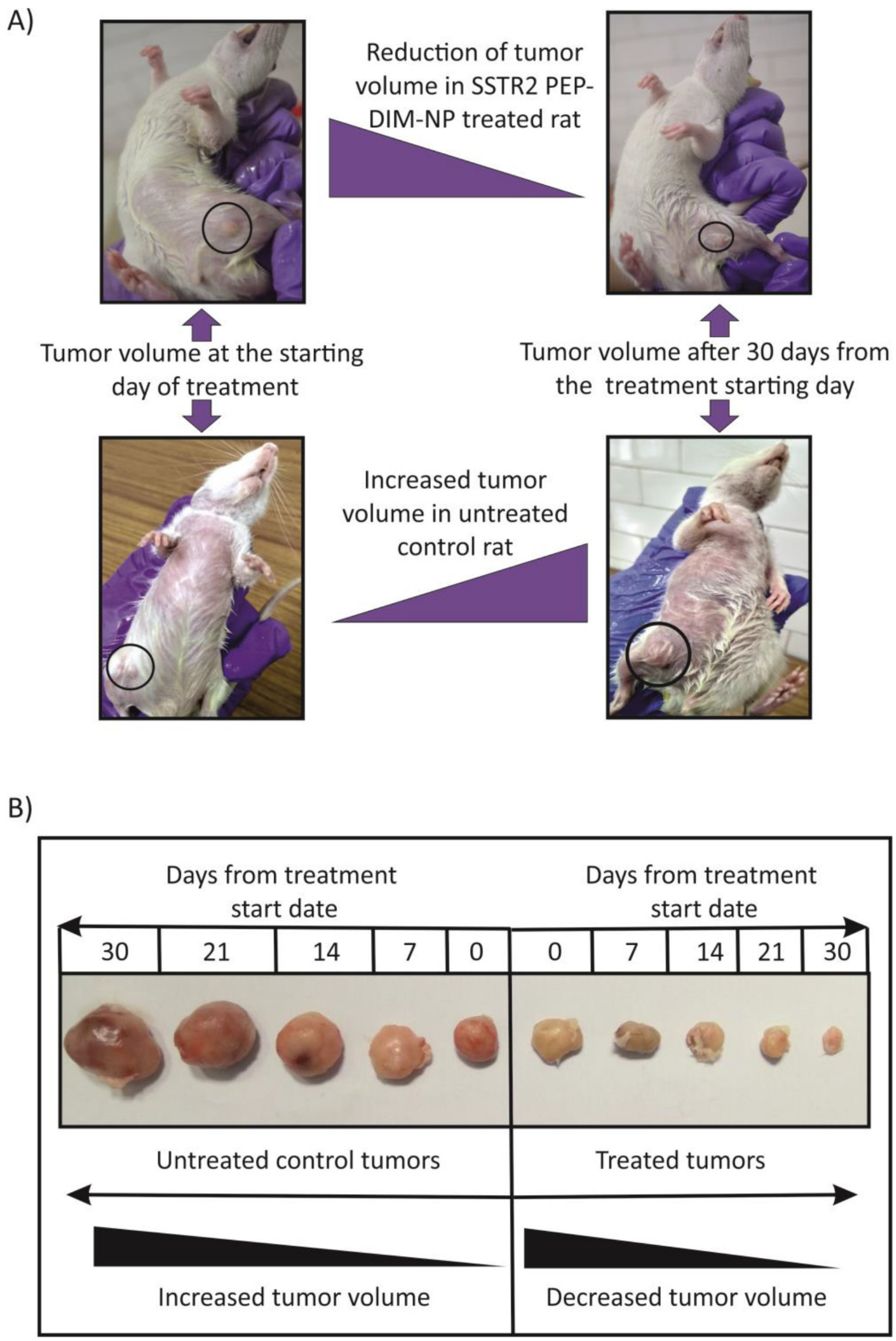

Supplementary Figure 8: Assessment of tumor volume reduction due to SSTR2 pep-DIM-NP treatment. (A) Figure represents growth inhibition of C6 tumor in same rat after nanoformulation treatment. In control untreated rat, tumor volume increased after 30 days. **(B)** Figure shows decreased volume of isolated treated C6 tumors at weekly intervals of treatment regimen. Control groups represent weekly increase in tumor volume. Rats were taken with almost same volume of tumors for both untreated and treated groups at the day 0 of treatment regimen.

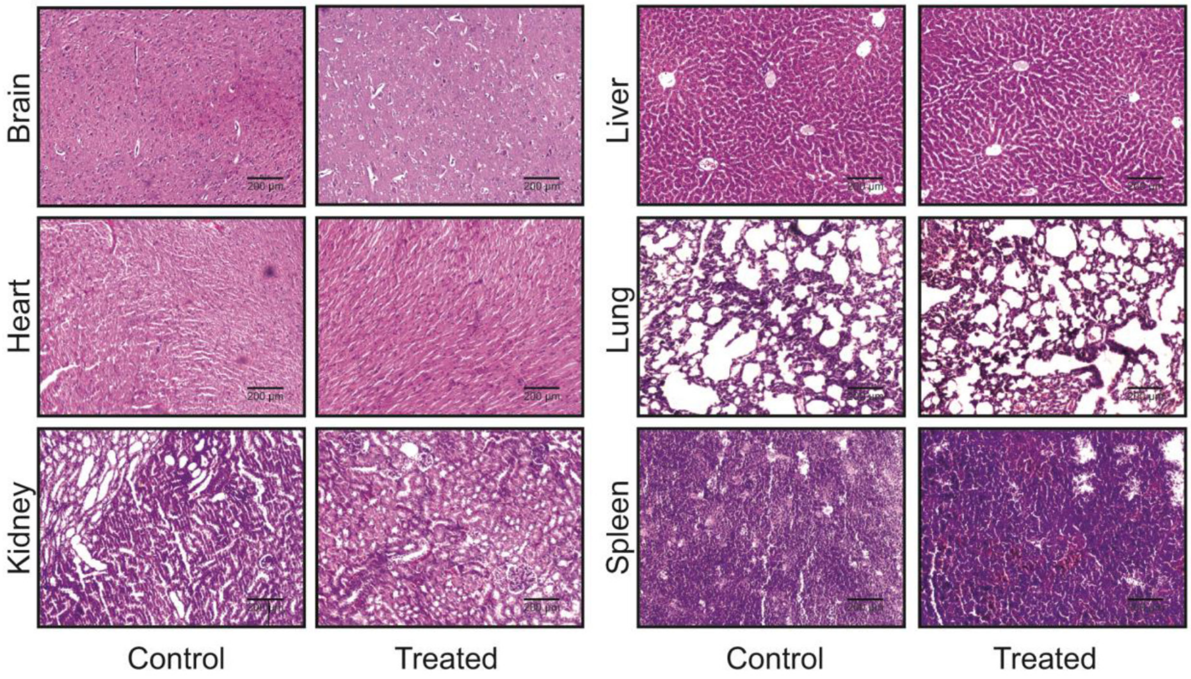

Supplementary Figure 9: *In vivo* internalization study of SSTR2 pep-DIM-NP. Images represent histology of body parts of untreated control and SSTR2 pep-DIM-NP treated SD rats.

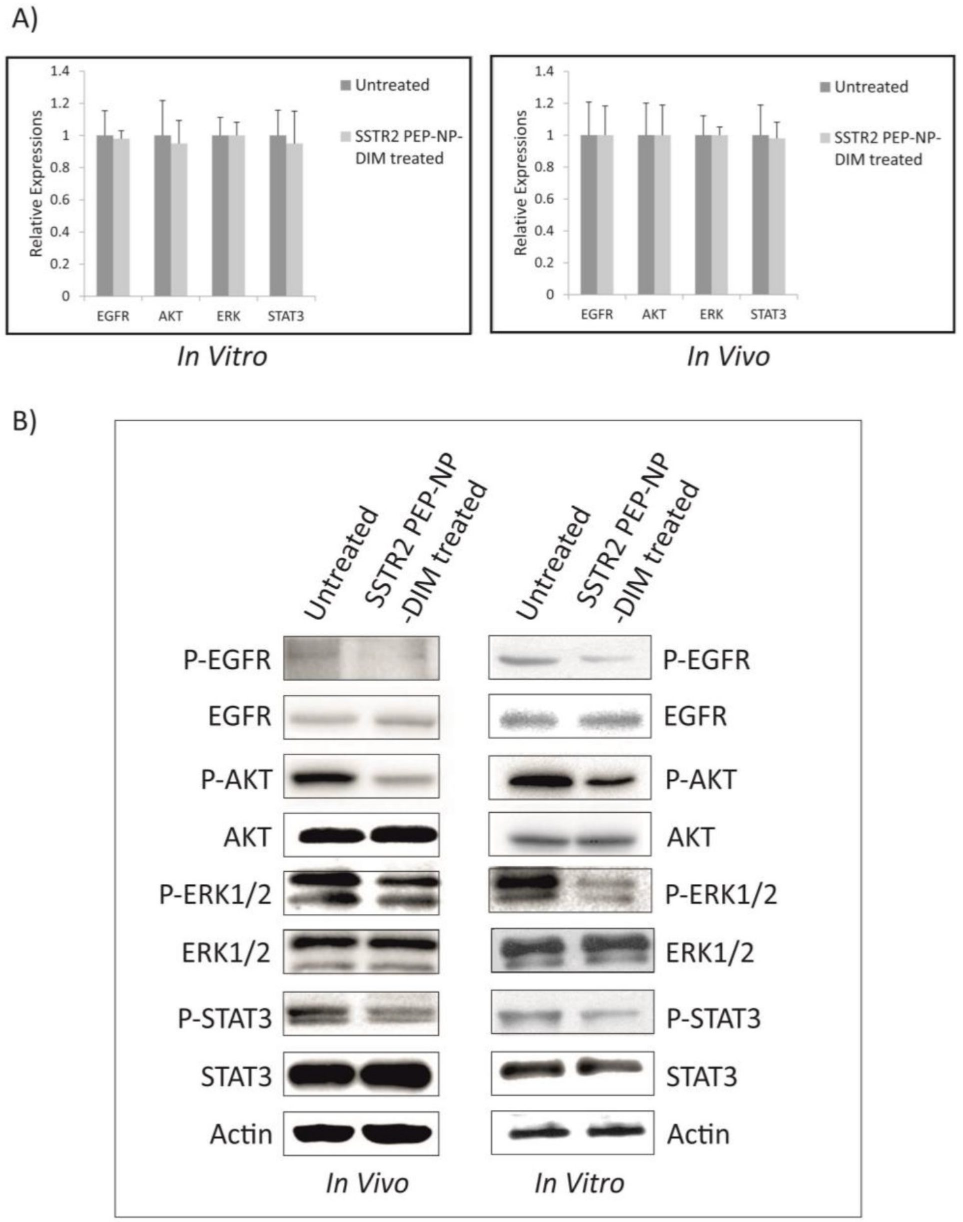

Supplementary Figure 10: Study of expression and activation of EGFR pathway members. (A) Total RNA extracted from nanoformulation treated and untreated C6 cells and C6 cell derived rat brain tumors were analysed by qRT-PCR for Egfr, Akt, Erk1/2 and Stat3. **(B)** Western bolt analysis represents expression and activation of EGFR pathway molecules *in vivo* (left panel) and *in vitro* (right panel) in treated and untreated groups.

Supplementary Table 1: Optimization of DIM loaded PLGA nanoparticles

S. No.	Batch no.	Drug-polymer ratio	Surfactant conc. (%w/v)	% Entrapment efficiency	Particle size (nm)
1	DIM-NP1	1:2	1	35.42% ± 0.26	350-400
2	DIM-NP2	1:2	2	44.61% ± 0.29	250-270
3	DIM-NP3	1:4	1	52.45% ± 0.30	190-235
4	DIM-NP4	1:4	2	60.66% ± 0.44	150-185
5	DIM-NP5	1:5	1	66.32% ± 0.43	110-140
6	DIM-NP6	1:5	2	75.20% ± 0.49	27-87

Values are expressed in mean±SEM, n=3.

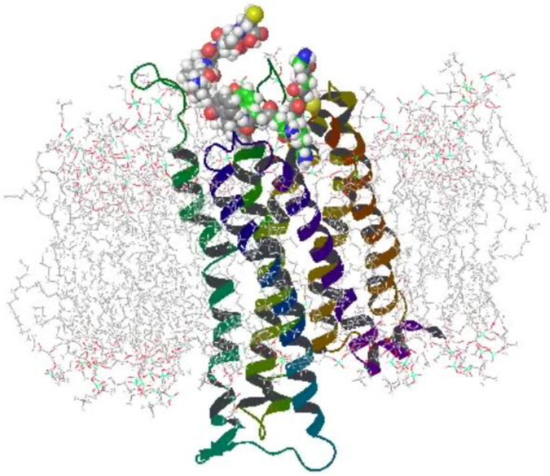

Supplementary Video 1: The closed conformation of the SSTR2 bound peptide at 3.1 ns indicated high stability and binding affinity even in the mimicry of biological conditions.

See Supplementary Video 1

Supplementary Table 2: Effect of temperature on % drug content, particle size and zeta potential of optimized DIM-NPs (A) and SSTR2 pep-DIM-NPs (B)

A)

S. No.	Time (in days)	Drug content (%) at 4±2°C	Drug content (%) at 25±2°C (RT)	Particle size (nm) at 4±2°C	Particle size (nm) at 25±2°C (RT)	Zeta potential (mV) at 4±2°C	Zeta potential (mV) at 25±2°C (RT)
1	0	98.67 ± 0.19	98.56 ± 0.24	27-87	27-87	65.0±8.35	65.0±8.32
2	15	98.35 ± 0.22	98.47 ± 0.35	27-87	35-85	64.0±8.31	65.0±8.43
3	30	97.55 ± 0.31	97.36 ± 0.27	35-85	40-85	64.0±7.29	63.6±8.37
4	60	96.85 ± 0.36	97.22 ± 0.39	35-85	40-85	64.5±6.71	63.4±8.47

Values are expressed in mean ± SEM, n=3. RT, Room Temperature.

B)

S. No.	Time (in days)	Drug content (%) at 4±2°C	Drug content (%) at 25±2°C (RT)	Particle size (nm) at 4±2°C	Particle size (nm) at 25±2°C (RT)	Zeta potential (mV) at 4±2°C	Zeta potential (mV) at 25±2°C (RT)
1	1	98.55±0.23	98.20±0.35	28-98	28-98	67.0±7.84	67.0±8.17
2	15	98.25±0.15	97.75±0.39	34-96	40-96	66.0±8.21	66.0±8.32
3	30	97.67±0.20	97.37±0.39	34-96	40-96	66.0±8.23	65.0±7.55
4	60	97.10±0.32	96.85±0.24	34-96	40-95	66.0±7.31	65.0±7.15

Values are expressed in mean ± SEM, n=3. RT, Room Temperature.
